# Differential proteomic profiling reveals regulatory proteins and novel links between primary metabolism and spinosad production in *Saccharopolyspora spinosa*

**DOI:** 10.1186/1475-2859-13-27

**Published:** 2014-02-21

**Authors:** Qi Yang, Xuezhi Ding, Xuemei Liu, Shuang Liu, Yunjun Sun, Ziquan Yu, Shengbiao Hu, Jie Rang, Hao He, Lian He, Liqiu Xia

**Affiliations:** 1 Hunan Provincial Key Laboratory of Microbial Molecular Biology-State Key laboratory Breeding Base of Microbial Molecular Biology, College of life Science, Hunan Normal University, Changsha 410081, China

**Keywords:** Proteomics, *Saccharopolyspora spinosa*, LC-MS/MS, Regulatory proteins, Spinosad production

## Abstract

**Background:**

*Saccharopolyspora spinosa* is an important producer of antibiotic spinosad with clarified biosynthesis pathway but its complex regulation networks associated with primary metabolism and secondary metabolites production almost have never been concerned or studied before. The proteomic analysis of a novel *Saccharopolyspora spinosa* CCTCC M206084 was performed and aimed to provide a global profile of regulatory proteins.

**Results:**

Two-dimensional-liquid chromatography-tandem mass spectrometry (LC-MS/MS) identified 1090, 1166, 701, and 509 proteins from four phases respectively, i.e., the logarithmic growth phase (T1), early stationary phase (T2), late stationary phase (T3), and decline phase (T4). Among the identified proteins, 1579 were unique to the *S. spinosa* proteome, including almost all the enzymes for spinosad biosynthesis. Trends in protein expression over the various time phases were deduced from using the modified protein abundance index (PAI), revealed the importance of stress pathway proteins and other global regulatory network proteins during spinosad biosynthesis. Sodium dodecyl sulfate-polyacrylamide gel electrophoresis analysis followed by one-dimensional LC-MS/MS identification revealed similar trend of protein expression from four phases with the results of semi-quantification by PAI. qRT-PCR analysis revealed that 6 different expressed genes showed a positive correlation between changes at translational and transcriptional expression level. Expression of three proteins that likely promote spinosad biosynthesis, namely, 5-methyltetrahydropteroyltriglutamate-homocysteine S-methyltransferase (MHSM), glutamine synthetase (GS) and cyclic nucleotide-binding domain-containing protein (CNDP) was validated by western blot, which confirmed the results of proteomic analysis.

**Conclusions:**

This study is the first systematic analysis of the *S. spinosa* proteome during fermentation and its valuable proteomic data of regulatory proteins may be used to enhance the production yield of spinosad in future studies.

## Background

*Saccharopolyspora spinosa* is a Gram-positive actinomycete which was originally isolated from soil [1] and can produce spinosad as a secondary metabolite. Spinosad contains the spinosyns A and D, produced by fermentation and used as a novel environment-friendly agent for insect control [2]. Spinosyns are produced by a polyketide pathway, but differs from the more common type I polyketides because it contains three intramolecular carbon–carbon bonds [3]. Several molecular biology-based methods have been used to over express genes that directly participate in spinosad biosynthesis to efficiently improve the spinosad yield [4]. However, the production of antibiotic is controlled by both pathway-specific and global regulators, such as transcription factors and signaling molecules. Few studies have focused on the regulatory pathways and networks of spinosad production in *S. spinosa*, possibly because none of the strains had their genome sequenced until 2011 [5]. Evidence shows that secondary metabolites are not uniquely expressed in the late stationary phase during nutrient limitation [6]. For example, the production of a type I polyketide is induced during the logarithmic phase in *Streptomyces coelicolor*[7]. Thus, a change in the physiological status of bacteria is probably not the main condition required to activate the spinosad biosynthesis pathways.

Proteome analysis has been widely used to identify enzymes or regulatory proteins that are associated with the production of antibiotics and the development or differentiation in bacteria [8]. More traditional methods for separating protein mixtures include two-dimensional gel electrophoresis (2D-GE) or two-dimensional fluorescence-difference gel electrophoresis (2D-DIGE), followed by enzymatic digestion and mass spectrometry (MS) [9,10]. Another popular method uses gel-enhanced liquid chromatography-tandem mass spectrometry (LC-MS/MS), wherein proteins in a mixture are separated by sodium dodecyl sulfate-polyacrylamide gel electrophoresis (SDS-PAGE) coupled with LC-MS/MS and label-free semi-quantification [11] or isobaric tags for relative and absolute quantitation (iTRAQ) [12]. Although 2D-GE (or 2D-DIGE) and gel-enhanced LC-MS/MS are superior in terms of their reproducibility and accurate quantification [13], these approaches have limitations for the detection of low-abundance proteins, hydrophobic proteins, and proteins with extreme sizes or charges [14]. High-performance liquid chromatography (HPLC) coupled with LC-MS/MS is a method that seems to have overcome these drawbacks; in this method, peptides are pre-fractionated by multidimensional chromatography and then detected by MS [15]. Thus, we used a strong cation exchange (SCX) column and reversed-phase (RP) separation coupled with MS/MS to analyze the proteome of *S. spinosa*.

In this study, we performed a detailed proteomic analysis of the spinosad-producing *S. spinosa* CCTCC M206084, which was isolated from a soil sample from a sugarcane plantation in Hunan Province (China). The analysis was conducted using 2D LC-MS/MS of four growth phases, namely, the logarithmic growth phase (T1), early stationary phase (T2), late stationary phase (T3), and decline phase (T4). Then we used a modified label-free semi-quantitative approach to estimate the trends in the varied abundance of protein expression. That is, the total number of identified peptides was normalized using the theoretically observable number of peptides and this ratio was defined as the protein abundance index (PAI) [16]. This study aimed to reveal the differences in the *S. spinosa* proteome during the fermentation process, including the identification of enzymes for spinosad biosynthesis and other regulatory proteins, such as stress proteins and metabolic switches. Thus, we created a database of protein expression profiles during the different growth phases of *S. spinosa* to facilitate further analysis of the regulation of these complex events, especially the regulatory pathways and networks of spinosad production.

## Results and discussion

### Growth phases and spinosad production analysis

**Figure 1:**
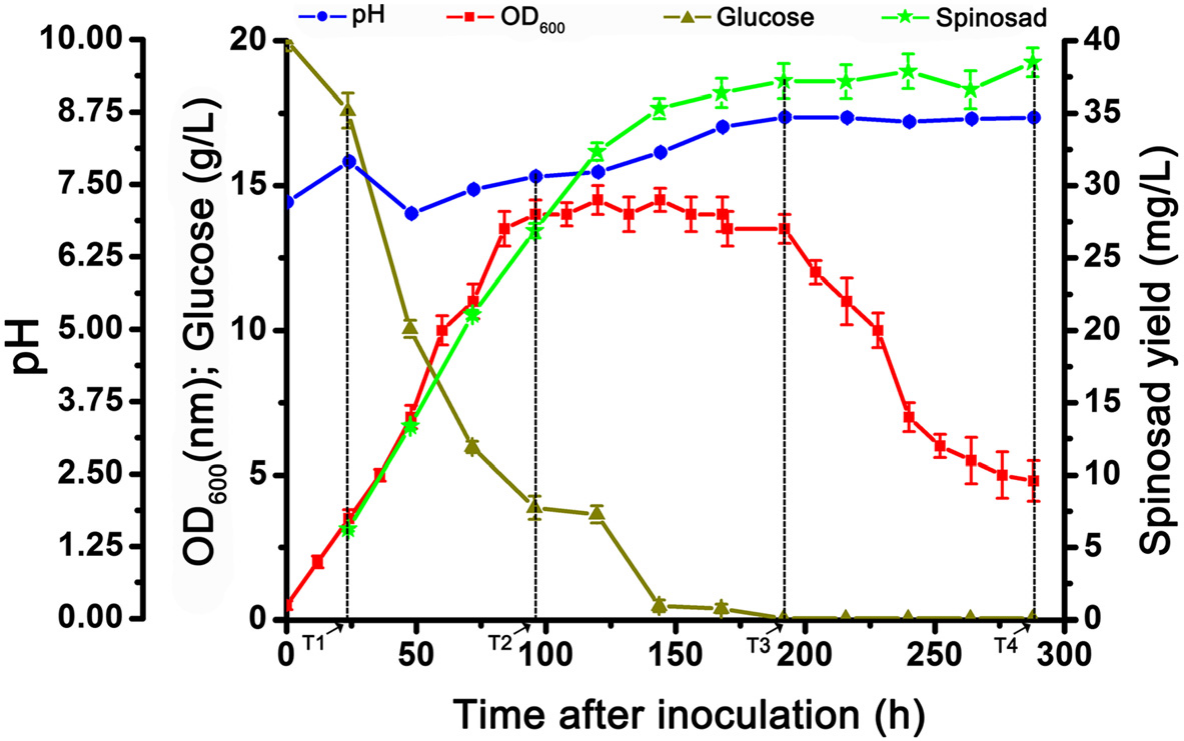
**Growth parameters of the *S. spinosa *CCTCC M206084 fermentation culture.** The growth curve (red line) was drawn according to the OD_600_ values, whereas the spinosad yield curve (green line) and glucose consumption curve (brown line) were drawn according to the analysis by HPLC, and the pH value (blue line) were taken to monitor growth every 24 h. Samples for proteomic analysis were collected at 2, 4, 8, and 12 d post-inoculation from fermentation cultures, as T1, T2, T3, and T4 phases, respectively. The logarithmic phase was from 0 d to T2, the stationary phase was from T2 to T3, whereas the decline phase was from T3 to T4.

Cell concentrations under OD_600_, glucose concentration in medium, pH value variation trend and spinosad production yield were monitored during bacterial cultivation (Figure 1). Cells quickly entered the logarithmic phase, with almost no distinct lag phase after inoculation. This rapid growth was maintained at the logarithmic phase for approximately 96 h (nearly 4 d). Compared with the long logarithmic phase, *S. spinosa* had a relatively short stationary phase and entered the decline phase with significant speed. The growth of *S. spinosa* was in agreement with the utilization of glucose which was reduced during the logarithmic phase and was almost exhausted in the stationary phase. Spinosad was detected almost throughout the entire incubation period of *S. spinosa* (12 d), the synthesis of spinosad stopped at the late stationary phase and no spinosad degradation occurred thereafter. The detection of spinosad at the logarithmic phase revealed that the production of microbial secondary metabolites was not unique to the late stationary phase which has been demonstrated in *S. coelicolor* and *Amycolatopsis balhimycina* before [6,8].

### Profiling of *S. spinosa* proteome

**Figure 2:**
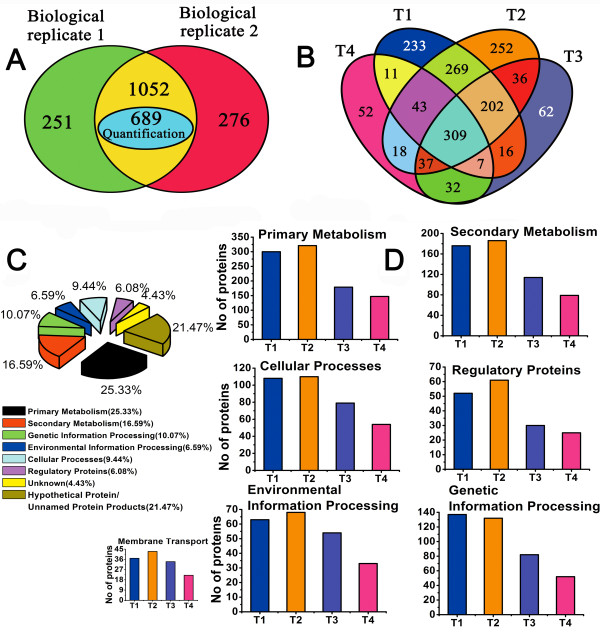
**Identification and functional description of the total proteins. (A)** Venn diagram showing the common proteins identified in the two biological replicates. The number of significant proteins was quantified, which had at least two unique peptides in each replicate. **(B)** Venn diagram of the protein distribution during different time phases. **(C)** Functional description of the *S. spinosa* proteome. **(D)** Number of proteins for each functional category.

To study the metabolic switches and adaptations deduced from the proteome of *S. spinosa* grown in batch culture, four time points were selected for proteomic analysis, according to its growth curve and spinosad production yield curve, that were 2, 4, 8, and 12 d, respectively (Figure 1). The number of proteins identified and quantified is shown in Figure 2A. A total of 1579 proteins were identified and represented 19.1% of the theoretical *S. spinosa* proteome, 1303 and 1328 proteins were identified in two biological replicates respectively. Approximately 67% of the proteins (1052 proteins) were present in both replicates, which reflected the high recapture rate between different biological replicates. Among the recaptured proteins, 689 were quantified by PAI value (Additional file 1: Dataset S1). 1090, 1166, 701, and 509 proteins were detected at each time point (2, 4, 8, and 12 d) respectively (Figure 2B). Certain proteins were identified in a single time point, whereas others were detected in multiple time courses. A total of 309 proteins were present in all four time points, 289 proteins were present in three time points, 382 were present in two time points, and 599 proteins were identified in only one time point (Figure 2B).

The identified proteins were classified based on their physical-biochemical characteristics, such as theoretical molecular weight (MW) and isoelectric point (pI) (Additional file 2: Figure S2). The detected proteome has proteins with predicted molecular masses ranging from 3.53 kDa (gi348175110, 30S ribosomal protein S18) to 781.36 kDa (gi41350159, ObsC) and pIs from 4.02 (gi348171622, acyl carrier protein or ACP) to 12.06 (gi348169208, histone-like DNA-binding protein) pH units. These ranges imply the extensive coverage of proteins with various physical properties (Additional file 1: Dataset S2). Thus, this data set represents the most complete inventory of the *S. spinosa* proteome, to date. Most of the identified proteins have a theoretical MWs ranging from 10 kDa to 80 kDa and pIs from 4 to 7 pH units. Therefore, a large percent of *S. spinosa* proteome are acidic proteins, while only a few have an extreme MWs. The percentage distribution of the identified proteins in each time point were not significantly different, except for the observed increase at T4 in the MWs ranging from 50 kDa to 60 kDa.

**Figure 3:**
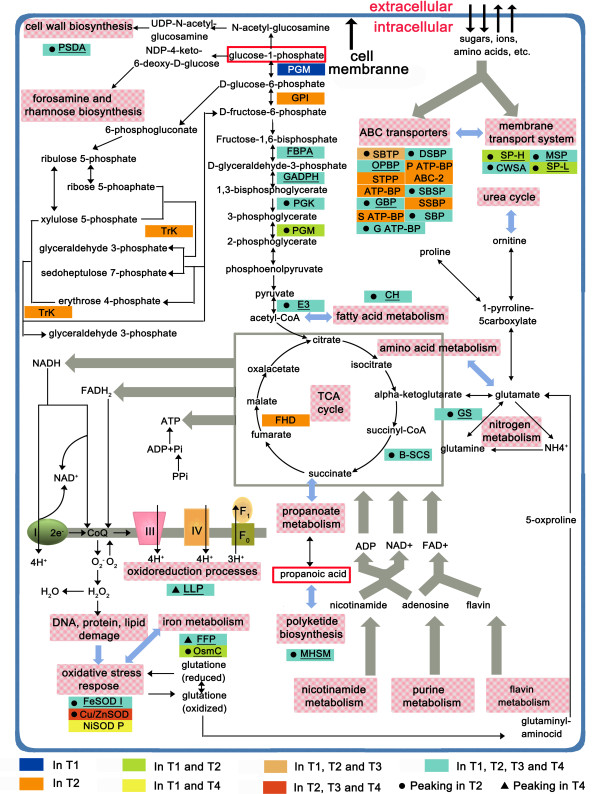
**Scheme of metabolic pathways where enzymes upregulated in specific growth phase during antibiotic production are highlighted.** Enzymes related to the synthesis of spinosad precursors were in red rectangles; the enzymes were underlined when the total PAI value more than 3 which revealed it is a abundant protein in *S. spinosa* proteome. Black dot indicated a enzyme owned a peak in T2 when compared to other three phases; black triangle signified a protein up-regulated in T2, T3 and T4 while compared to T1 and had a peak in T4. Reactions are reported according to KEGG metabolic pathway databases and Gallo et al. [10]. Enzyme name abbreviations refer to Additional file 3: Table S5.

Proteins were then grouped according to their putative functions (Figure 2C). The categories shown are primary metabolism, secondary metabolism, genetic information processing, environmental information processing, cellular processes and regulatory proteins. Most of the identified proteins were involved in primary metabolism (25.33% of the total) or hypothetical/unnamed protein products (21.74% of the total), the high percentage of hypothetical/unnamed protein products were in case of that those proteins have no homologues in other microorganism. Proteins for secondary metabolism had the third highest percentage (16.59% of the total), therefore, the biosynthesis of spinosad and other secondary metabolites accounted for a large proportion of *S. spinosa* metabolism. The numbers of proteins detected in four time phases were then compared according to their functional classification to understand the changes in protein numbers (Figure 2D; Additional file 1: Dataset S2). The total number of proteins in each time phase was progressively decreased from T1 to T4. Membrane transport proteins, which are included in a larger category named environmental information processing, play a major role in transportation of diffusible compounds like sugars, amino acids, peptides, signaling molecules and ion. Those proteins may be consistent with the requirement of some nutrients. The number of transporters had the highest accumulation rate at T2 and continued to accumulate until T3 which may reflect a greater requirement of nutrition in earlier stationary phase (Figure 2D). Three proteins involved in membrane transport system were detected at high abundance, including a membrane-spanning protein (gi348174826, MSP) and two secreted proteins (one with higher abundance, gi348173142, SP-H; another with lower abundance, gi348169435, SP-L), they all significantly up-regulated at T2 phase while compared to T1 (Figure 3). In addition, a cell wall surface anchor family protein (gi348176568, CWSA) in this transport system owned the same trends with these three abundant proteins. The accumulation of ABC transporters not only suggested an increased flux through cell membrane or a particular requirement of nutrients or diffusible compounds, but also may have some important functions during the transport of these secondary metabolites to extracellular sites so that spinosad could be detected in the fermentation supernatant when it was produced [17]. In *S. spinosa* proteome, twelve ABC transports had a peaking or were only identified at T2 phase, and two of them were detected at high abundance (Figure 3). The putative glutamate-binding protein (gi348171412), which is important in the ABC transporter system [18], was highly abundant and its expression was significantly increased at the whole stationary phase. As a periplasmic amino acid-binding protein, this protein not only relates to the use of medium peptide but also functions in the presence of the branched-chain amino acid aminotransferase and the oligopeptide-binding protein of the ABC transporter that recycles amino acids and peptides from lysed mycelia. In short, the obtained global protein expression profiles enhanced the overall understanding of the *S. spinosa* proteome.

### Stress-response and redox balance proteins

Some highly expressed proteins were stress-response and redox balance proteins that function as regulators during fermentation, and most of these proteins were significant upregulated in the T2 phase except the expression of five cold shock-related proteins (Figure 3; Additional file 3: Table S2). A member of the ferritin family protein (gi348175373, FFP) functioned in iron ion transport and involved in iron metabolism was found to promote DNA protection from oxidative stress and the nucleoid condensation during stationary phase [19], which was significantly upregulated in T2 while compared to T1 and then had a peaking in T4 while compared to other three time phases. The same trend was observed for the luciferase-like protein (gi348176208, LLP), which has a physiological function in the protection of cells against oxidative stress [20]. Superoxide dismutases (SODs) are ubiquitous enzymes that efficiently catalyze the dismutation of superoxide radical anions to protect biological molecules from the oxidative damage induced by oxidative stress [21]. In this study, though only FeSODI (gi348173178) demonstrated relatively high abundance, a total of three SODs were detected, including a nickel-containing SOD precursor (gi348168941, NiSOD P) and the SOD copper/zinc-binding protein (gi348174946, Cu/ZnSOD). Among them, FeSODI and Cu/ZnSOD were all up-regulated at T2 phase while compared to T1. Studies showed that the expression of SODs is repressed by feedback inhibition, the SOD activity was positively correlated with the amount of each protein [22], which illustrates the enormous difference in the protein expression abundance of the three SODs. Thus, we inferred that FeSODI is a key protein for protecting *S. spinosa* cells from oxidative stress, as compared with the other two SODs. Interestingly, FeSOD had an abundance level not depending on oxygen in *K. oxytoca* BAS-10 [23] and evidence suggests that FeSODI can enhance secondary metabolite production during fermentation as first studied in *Streptomyces clavuligerus* and *Streptomyces lividans* TK24 [24], so we supposed that FeSODI may be a key player of the biosynthesis of spinosad. Regulators of osmotic stress are more common during the transition or early stationary phases [25], a protein from the OsmC family (gi348168991, OsmC) was significantly upregulated in the T2 phase while compared to other three growth phases after its induction by osmotic stress. Furthermore, the prokaryotic ubiquitin-like protein Pup (gi348173932) is a stress protein functionally analogous to Ub in *Mycobacterium tuberculosis* and directs proteins to the proteasome, which may be accumulated as the fermentation progressed when carbon or nitrogen source become limiting in the medium [26,27]. Pup was detected in low amounts at T4 phase as an important stress regulatory protein in *S. spinosa* and revealed a general response to the nutrient limitation in decline phase.

### Proteins in primary metabolism

Most differently expressed and higher abundant proteins were involved in glucose consumption, including glycolysis, TCA cycle and pentose phosphate pathway (Figure 3; Additional file 1: Dataset S1). In particular, glycolytic enzymes glucose-6-phosphate isomerase (gi348176000, GPI), phosphoglycerate kinase (gi348174018, PGK), phosphoglycerate mutase (gi348174887, PGM) and dihydrolipoamide dehydrogenase (gi348171500, E3) were up-regulated or just detected in T2 while compared to other three growth phases. On the other hand, fructose-bisphosphate aldolase (gi348175040, FBPA) and glyceraldehyde 3-phosphate dehydrogenase (gi348174019, GAPDH) were abundant proteins in *S. spinosa* proteome though without a peaking at T2. The upregulation and higher abundance of these enzymes suggests an increased metabolic flux throughout glycolysis. This result is in agreement with the upregulation of the enzyme transketolase (gi348175998, TrK) belonging to non-oxidative branch of pentose phosphate pathway which was just identified at T2 phase. In addition, another enzyme involved in glucose metabolism, phosphoglucosamine mutase (gi348176848, PGM), functioned in transforming the glucose-6-phosphate to the spinosad precursor glucose-6-phosphate was just identified in T1 phase which may give a good preparation for spinosad biosynthesis. The upregulation of glycolytic enzymes is also in agreement with the trend of TCA cycle enzymes succinyl-CoA synthetase subunit beta (gi348176767, B-SCS) and fumarate hydratase (gi348170953, FHD), which owned a peaking in T2 (Figure 3). These findings were correlated with the growth curve of *S. spinosa,* production yield of spinosad and the rate of glucose consumption (Figure 1) which suggested that the limitation of glucose may be an important factor to induce the transition of growth phases in *S. spinosa*. Two proteins induced by starvation (gi348172896, gi348170958) were identified in *S. spinosa* at low abundance. Among these proteins, a starvation-response DNA-binding protein was quantified by its PAI and remarkably upregulated in late stationary phase and decline phase while compared to logarithmic phase (Additional file 1: Dataset S1).

Besides a large percents of proteins related to glycolytic enzymes, some others with significantly up- or down-regulation were also involved in primary metabolism. Choloylglycine hydrolase (gi348170064, CH) participated in fatty acid metabolism, glutamine synthetase (gi348171511, GS) functioned in nitrogen metabolism and polysaccharide deacetylase (gi348174074, PSDA) effected on cell wall biosynthesis were all high abundant proteins and showed the highest accumulation during the earlier stationary phase (T2) (Figure 3). According to studies carried out in *A. balhimycina* showing that during growth phases glutamate was consumed as primary carbon and nitrogen source causing a pH increment [28], the trend of GS in *S. spinosa* may reflect the intense requirement of glutamate and the pH value increased from 7.2 to 8.67 (Figure 1). The PSDA which was first discovered in *Bacillus subtilis,* participates in carbohydrate metabolism as well as regulates cell wall formation that has a positive effect on sporulation and may function as an *N*-acetylmuramic acid deacetylase in *B. subtilis*[29,30]. The relative abundance of PSDA had peaked at T2 phase during *S. spinosa* fermentation, may indicate that cell wall formation and bacteria growth peaks at the onset of the stationary phase (Figure 3).

### Global regulators in *S. spinosa*

**Figure 4:**
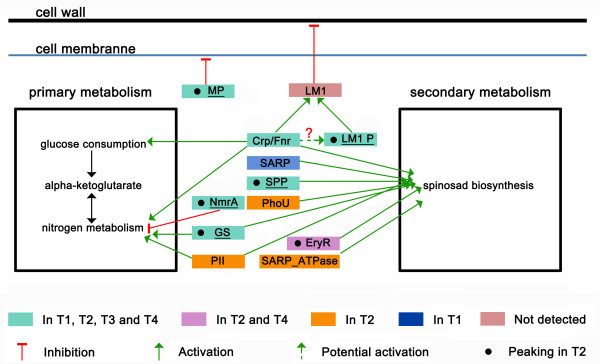
**Overall illustration of global regulators between primary metabolism and secondary metabolism in *S. spinosa*.** Enzymes were underlined when the total PAI value more than 3 which revealed it is a abundant protein in *S. spinosa* proteome. Black dot indicated a enzyme owned a peak in T2 when compared to other three phases. Enzyme name abbreviations refer to Additional file 3: Table S5.

In agreement with the increased expression of most primary metabolism enzymes in T2, some upregulated proteins with the same trend are global regulators in *S. spinosa* (Figure 4). Proteins participated in the regulation of nitrogen metabolism were mostly up-regulated at T2 phase. GS participates in amino acid metabolism and functions as a metabolic switch during nitrogen assimilation by targeting GlnR [31,32]; and the overexpression of an antisense non-coding RNA targeting GS decreases the production of ectoine in *S. coelicolor*[33]. Therefore, evidence showed that GS may be a key regulatory protein for spinosad biosynthesis. Other regulators NmrA family (gi348173307, NmrA) and PII (gi348169280, PII) were also involved in nitrogen metabolism, NmrA plays a role of repression [34] while PII functioned in promotion [35,36]. The transcriptional regulator from the Crp/Fnr family (gi348172925, Crp/Fnr) is a central regulatory protein in the life cycle of *S. coelicolor* and the secondary metabolism while Crp overexpression enhances the production of antibiotics [37,38]. In particular, Crp/Fnr is a catabolite repressor protein related to the glucose exhaustion while the glucose concentration was limited during growth and also could be up-regulated under N-limitation in the medium as studied in *K. oxytoca* BAS-10 and *E. coli* respectively [23,28]. The accumulation of Crp/Fnr were correlated with the consumption of glucose (Figure 1) and reflected a strong requirement of nutrient since early stationary phase. Besides, Crp may promote the expression of lysozyme M1 which is primarily involved in the control of cell wall thickness as studied in *S. coelicolor*[37]. Although lysozyme M1 was not detected in our study, three lysozyme M1 precursor proteins were identified and one of these proteins (gi348170152, LM1 P) was highly abundant in *S. spinosa* and rapidly increased during the T2 phase. However, the correlation between Crp/Fnr and LM1 P was unclear at present and we put forward an hypothesis that Crp/Fnr possibly have positive effect on the expression of LM1 P (Figure 4). In addition, an identified metallopeptidase (gi348174902, MP) functions in catabolism and cell division by degrading a set of short-lived proteins to disrupt membrane stability [39], the upregulation of this protein may affect the stability of cell membrane and also account for the decreased number of proteins during the last two phases. A number of proteins related to spinosad biosynthesis were also identified and mostly have a peaking at T2 phase. SARP family (gi348172759, SARP_ATPase; gi348170377, SARP) represents a unique regulatory mechanism that controls the biosynthesis of bioactive secondary metabolites in *Streptomyces* species [40]. Moreover, the PhoU-related protein (gi348174954, PhoU) is a phosphate transport system regulator and involved in the general mechanism governing secondary metabolism [41]. The serine protease precursor (gi348173083, SPP), a signal transduction protein involved in the two-component system and evidently increased at the T2 phase, is a general key player in secondary metabolism in *S. coelicolor*[42]. A pleiotropic regulatory protein EryR (gi346655273), which is 77% identical to BldD of *S. coelicolor* and 99% to the model strain from *Saccharopolyspora* (*Saccharopolyspora erythraea* strain NRRL2338) respectively, peaked in T2 time phase and may positively promote the spinosad production in *S. spinosa* while *S. erythraea bldD* mutants produce a bald phenotype and 7-fold less erythromycin than the *S. erythraea* wild type strain [43]. Those proteins perhaps were key players or regulators in the spinosad biosynthesis and, to the best of our knowledge, this paper is the first report of such a regulatory networks for secondary metabolite biosynthesis in *S. spinosa*.

### Proteins for spinosad biosynthesis

**Figure 5:**
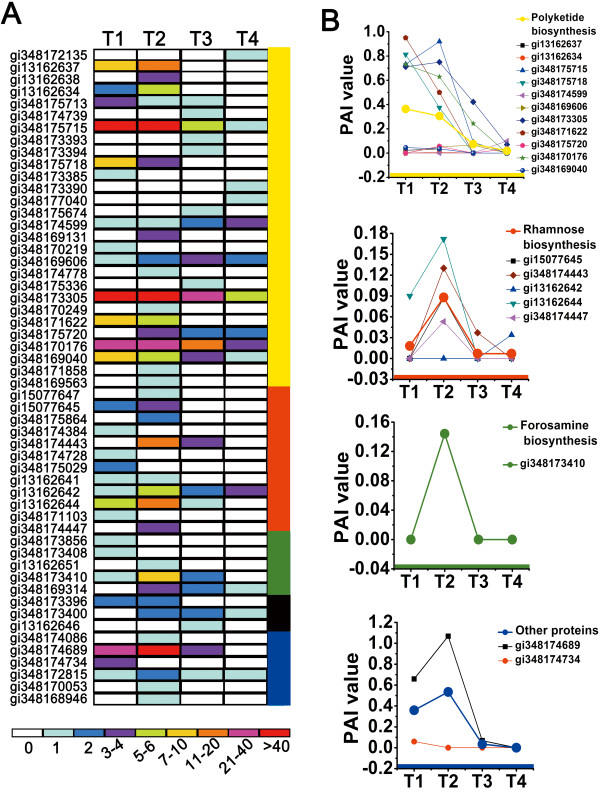
**Spinosad biosynthesis enzymes detected in the total proteome. (A)** Number of identified peptides for the 54 proteins that participated in spinosad biosynthesis. The proteins were subdivided into five clusters according to their functions, as indicated by the colored bars on the right: polyketide biosynthesis, rhamnose biosynthesis, forosamine biosynthesis, polyketide bridging, and other proteins. The total peptides of a unique protein was indicated at the bottom of the panel. **(B)** Abundance trends for the 19 semi-quantified proteins based on their PAI values, the dynamics of the individual proteins, and the average PAI of each functional cluster, which are shown on the right (the thick lines presented the an average PAI of all proteins in the different clusters).

The timing appearance of enzymes required for spinosad biosynthesis pathways were almost identified in this study (Figure 5; Additional file 3: Table S3). Most proteins related to spinosad biosynthesis could not be quantified because of their low abundance. Given that the number of peptides was closely related to the relative protein abundance, we used the number of peptides to estimate the dynamic behavior of a unique protein during fermentation. A heat map (Figure 5A) was constructed according to the data of 54 proteins directly related to spinosad biosynthesis. These enzymes were classified into five clusters, namely, polyketide biosynthesis, rhamnose biosynthesis, forosamine biosynthesis, polyketide bridging, and other proteins possibly participating in spinosad biosynthesis. Each cluster corresponded to a specific colored column on the right of the heat map. The yellow column shows the involvement of 28 proteins in polyketide biosynthesis, seven modular polyketide synthases, one loading module encoded by the *spnA* gene, and six extender modules (Additional file 3: Table S3). The full complement of the domains required for polyketide biosynthesis was detected, including ketoacyl synthase, acyltransferase, ketoacyl reductase, ACP, enoyl reductase, hydroxyacyl dehydratase, and carboxy-terminal thioesterase. The red column stands for 10 rhamnose biosynthesis proteins encoded by the *gdh*, *gtt*, *spnH*, *spnI*, and *spnK* genes, whereas the *spnG* gene encodes a rhamnosyltransferase [44]. Three different glycosyltransferases were identified in *S. spinosa* proteome after a BLAST search of the detected unique peptides and sequence alignment of their amino acid sequences. An S-adenosyl-l-methionine (SAM)-dependent methyltransferase (MT) (gi348171103) was identified in the T1 phase based on its unique peptide, except for the three O-MTs encoded by *spnH*, *spnI*, and *spnK*, respectively. The *spnH*, *spnI*, and *spnK* genes had high sequence identity with those encoding the SAM-dependent MTs involved in the O-methylation of rhamnose [45]. Thus, this novel SAM-dependent MT may be involved in rhamnose biosynthesis. The genes for NDP-glucose synthase and NDP-glucose dehydratase were within the same cluster; these enzymes are required for the conversion of glucose-1-phosphate to NDP-4-keto-6-deoxy-d-glucose, which is a common intermediate in the biosynthesis of 6-deoxysugars such as rhamnose [46]. A unique UDP-glucose/GDP-mannose dehydrogenase (gi348174447) was detected in the T2 phase and may contribute to spinosad biosynthesis. The green column contains five proteins for forosamine biosynthesis. Three of these proteins (gi348173408, gi13162651, and gi348173410) were confirmed to be encoded by the *spnQ*, *spnR*, and *spnS* genes, respectively. The *spnN* gene encodes an oxidoreductase domain-containing protein in *S. spinosa*, which was not detected in the present study. However, another oxidoreductase domain-containing protein (gi348173856) was identified instead, which may have the same function as the previously reported protein [3]. A PLP-dependent-4-aminobutyrate aminotransferase (gi348169314) coded by the *spnR* gene was detected after the T1 phase [47], which may participate in forosamine biosynthesis (Figure 5A). Three polyketide-bridging proteins encoded by *spnF*, *spnJ*, and *spnM* were all detected and marked with a dark green column. Six MTs, including O-MT and type 11 MT, were classified into the last group of proteins and labeled with a black column to show their close relationship with spinosad biosynthesis. Three transcriptional regulators from the lysR family encoded by the spinosad gene cluster ORF-L16 were detected in *S. spinosa* proteome though without quantification and these regulators were not treated as spinosad biosynthesis enzymes because they generally had no effect on spinosad production [48,49]. Although most enzymes for spinosad biosynthesis had low abundance, 19 proteins were still quantified (Figure 5B; Additional file 1: Dataset S1), with high reproducibility and accuracy during dynamic proteomic analysis. The up- or downregulation trends of the quantified proteins are shown in Figure 5B. Only four functional clusters were quantified, namely, polyketide biosynthesis, rhamnose biosynthesis, forosamine biosynthesis, and other related proteins. Eleven proteins for polyketide biosynthesis were quantified and their average PAI values (thicker line) followed similar trends. The abundance of these proteins was constant in T1 and T2 but downregulated in T3 and T4 while the other three functional clusters were all significantly upregulated in T2. Compared with other time phases, the variation of enzymes in the T2 phase follows a basic trend in spinosad biosynthesis, although only one quantified protein was observed in the forosamine biosynthesis cluster. All evidence indicates that the earlier stationary phase (T2) is the key phase for secondary metabolite biosynthesis in *S. spinosa*.

### Quantitative RT-PCR of key transcripts

**Figure 6:**
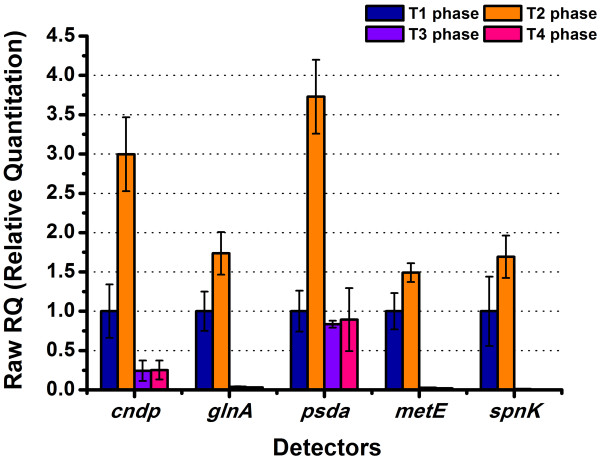
**qRT-PCR analysis of selected key transcripts.** qRT-PCR was used to substantiate differential expression patterns of five selected genes (*cndp*, *glnA*, *psda*, *metE* and s*pnK*). mRNA levels from four time phases are expressed as relative values to 16S rRNA, arbitrarily setting the ratio values for the T1 sample to 1. Error bars are calculated from four independent determinations of mRNA abundance in each sample.

To study if the changes in the proteome were correlated with differences at the mRNA level, the expression of five selected genes, essential for different metabolic processes, was analyzed by qRT-PCR. The corresponding five proteins were involved in nitrogen metabolism (GS), carbohydrate metabolism and cell wall biosynthesis (PSDA), spinosad biosynthesis (probable O-methyltransferase), as well as other two potential key players on spinosad production (MHSM and CNDP), respectively. Alignments of amino acid sequences from those proteins between *S. spinosa* and *S. erythraea* strain NRRL 2338 showed that GS and MHSM are encoded by genes *glnA* and *metE* respectively. However homologous amino acid sequences with high identity of PSDA and CNDP were almost not found, so *psda* and *cndp* were considered to represent the encoding genes of PSDA and CNDP temporarily in this study. qRT-PCR revealed that all these genes owned a peaking at T2 phase (Figure 6) and had similar trend with 2D-LC-MS/MS results, showed a positive correlation between changes at translational and transcriptional expression level.

### SDS-PAGE analysis and validation by Western blot

**Figure 7:**
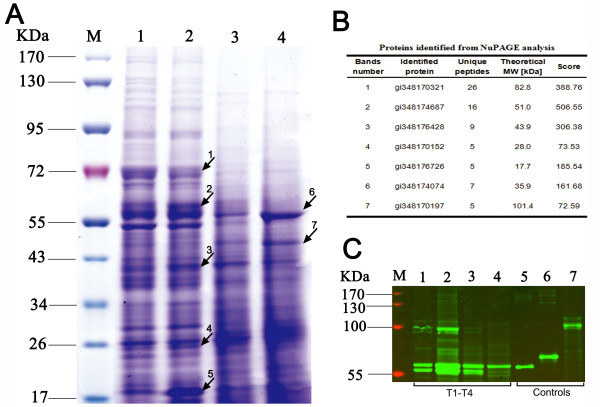
**SDS-PAGE gel analysis and Western blot validation of samples from the four time phases of *S. spinosa. *(A) **SDS-PAGE gel analysis. M: pre-stained protein marker; Lanes 1 to 4: protein samples from T1, T2, T3, and T4 of *S. spinosa* cells, respectively; Arrows 1 to 7: gel bands selected for 1D-LC-MS/MS identification. **(B)** A table list of proteins identified by 1D-LC-MS/MS. The protein with the highest score for a gel band was considered the corresponding protein of the said gel band. **(C)** Western blot validation of the abundance of CNDP, GS, and MHSM expression in *S. spinosa.* M: pre-stained protein marker; Lanes 1 to 4: protein samples from T1, T2, T3, and T4 of *S. spinosa* cells, respectively; Lanes 5 to 7: positive controls of CNDP, GS, and MHSM [the purified heterologous expression proteins].

The intensity of seven gel bands increased in T2 and T4 respectively, which revealed the corresponding proteins may be upregulated as compared with other time phases. Those bands were selected for gel-based 1D-LC-MS/MS identification to show a similar expression trend with the 2D-LC-MS/MS results (Figure 7; Additional file 2: Figure S3). The proteins detected in these bands owned the similar expression trends with label-free quantization, except for bands 6 and 7 whose theoretical MWs were greatly different from the SDS-PAGE results. Band 6 is higher and this may be consistent with covalently modifications (or covalent heterodimerization) that makes higher the MW, while band 7 is lower and this may be consistent with physiological degradation. In addition, western blot analysis was used to validate the expression of MHSM, GS, and CNDP in *S. spinosa* (Figure 7). These proteins were all detected throughout the entire fermentation process, and their expression was significantly upregulated in the T2 phase. These results conformed to those obtained by 2D LC-MS/MS.

## Conclusions

In summary, the highly detailed and comprehensive analysis of the *S. spinosa* proteome presented in this study has produced, to date, the most complete database of protein profiles during *S. spinosa* growth and spinosad biosynthesis in liquid fermentation cultures. Several important regulatory proteins or key players were significantly differentially expressed during fermentation. The detailed genetic and biochemical analyses of these proteins in future studies could provide valuable information on *S. spinosa* primary metabolism and spinosad production. The identification of the total proteome of *S. spinosa* is only a starting point for analyzing the regulatory network between bacterial primary metabolism and spinosad biosynthesis. Three potential regulatory proteins or key players (MSHM, GS, and CNDP), which were selected based on the results of 2D-LC-MS/MS, were needed more researches to study their regulatory mechanism on spinosad production. Studies on the knockout of their corresponding genes are useful to understand the control nodes and regulatory networks of bacterial primary metabolism and spinosad biosynthesis, then more valuable information will be obtained by comparative proteomic and metabolomic analysis between mutant and original strains.

## Methods

### Bacterial cultures

The *S. spinosa* CCTCC M206084 used in this study was isolated by our laboratory from the south of China (wild-type strain). The spores of bacteria was cultured in activation media containing glucose (10.0 g/L), trypticase soy broth (45.0 g/L), yeast extract (9 g/L), and MgSO_4_ · 7H_2_O (2.2 g/L) in a 225 mL flask, with a starting volume of 50 mL at 30°C and 300 rpm. After bacteria has been incubated in the activation medium for 48 h, a 2 mL aliquot of the seed culture was transferred into a 300 mL baffled flask containing 20 mL of the fermentation media and incubated on a humidified rotary shaker incubator (NBS, USA) at 30°C and 300 rpm with 70% relative humidity. The fermentation media (pH 7.2) contained KNO_3_ (1.0 g/L), FeSO_4_ (0.01 g/L), K_2_HPO_4_ · 3H_2_O (0.5 g/L), MgSO_4_ · 7H_2_O (0.5 g/L), glucose (20.0 g/L), yeast extract (4.0 g/L), and tryptone (4.0 g/L). To monitor the growth profile of *S. spinosa* in the batch cultures with the media, optical density at 600 nm (OD_600_) was used to determine the cell concentration during fermentation. Cells were collected every 12 h for growth curve measurements by UV scanning and collected every 24 h to determine the spinosad production and glucose concentration using an HPLC system (ÄKTA Purifier10; GE Healthcare, USA). The quantification of spinosad by UV-HPLC was performed as previously described with slight modification [4], spinosad was extracted from the suspension culture by incubating the cell suspension with acetone at a 1:1 volumetric ratio for 48 h. Cultures were centrifuged at 9000 rpm for 15 min, and the supernatants were filtered through 0.22 μm Millipore filters. After filtration, a 10 μL aliquot of each supernatant was loaded onto a C18 column (AQ12S05-1546WT) and eluted with the elution buffer at 1.5 mL/min. The elution buffer contained methanol, acetonitrile (ACN), and 2% aqueous ammonium acetate in a specific volumetric ratio (45:45:10). The detection wavelength was set at 250 nm during the analysis. Glucose concentration in medium was measured as previously described [8], that were analyzed by HPLC system equipped with an AMINEX HPX-87 H column (Bio-Rad Laboratories, Hercules, CA) and detected at 210 nm for quantification. The pH trend during fermentation was measured every 24 h.

### Preparation of whole cell proteins

Cells were harvested (9000 rpm, 10 min, 4°C) at four specific time points (2, 4, 8, and 12 d) and washed four times with a suspension in PBS (10 mM, pH 7.8) pre-chilled at 4°C. Cells were then quickly frozen with liquid nitrogen, transferred to a pre-chilled pestle, and ground into powder using a mortar and pestle. The cell powder (0.2 g) was placed in a sterilized Eppendorf tube at 4°C and suspended in lysis buffer (0.4 mL) containing 8 M urea, 2 M thiourea, 4% (w/v) CHAPS, 75 mM NaCl, 50 mM Tris-HCl (pH 8.0), 2 mM phenylmethylsulfonyl fluoride, and 4 μL of a protease inhibitor cocktail. The suspended matter was shaken and mixed on a vortex for 30 s at 20 min intervals for 2 h. The lysed cells were centrifuged at 4°C and 13 200 rpm for 45 min to obtain the cleared cell extract. The supernatant was then stored at -80°C until further use. Before MS analysis, the protein concentrations of the supernatants were determined using a 2-D Quant kit (Amersham Biosciences, Piscataway, NJ).

### Preparation of tryptic peptides for 2D-LC-MS/MS analysis

Samples were prepared from two biological replicates at four time points, thus, the entire data set was derived from eight experiments, i.e., each time point has two biological replicates. Proteins from cell lysates (300 ug of total protein) were volumed to 100 μL, using lysis buffer to adjust their concentrations to 3 μg/μL. Subsequently, 100 μL of 100 mM NH_4_HCO_3_ (pH 8.0) was added to each sample. The solution was reduced in a water bath at 37°C by adding 2 μL of 0.5 M dithiothreitol (DTT) and incubating the solution for 60 min. The samples solutions were then alkylated by adding 3 μL of 1 M iodoacetamide and incubating the cells in the dark for 60 min. The excess iodoacetamide was quenched by adding 15 mM to 20 mM DTT and incubating for 15 min. The urea concentration was then diluted from 4 M to 1 M using 800 μL of NH_4_HCO_3_ solution. The sample solutions were then incubated overnight with trypsin in a trypsin/Protein ratio of 1:50 (w/w) in a water bath at 37°C. Samples were further desalted and concentrated using an Oasis HLB sample cartridge column (Waters Corporation). The purified peptides were dried at 37°C by vacuum freeze-drying.

### SDS-PAGE and In-gel digestion

Proteins from cell lysates (20 μg of total protein of each phase) were added to lysis buffer to reach a concentration of 2.5 μg/μL and incubated in boiling water for 10 min with 2 μL of 4× sample loading buffer (Invitrogen). Proteins were separated on 4% to 12% NuPAGE gels (Invitrogen), stained with Coomassie brilliant blue (CCB) for 3 h, and destained overnight. The destaining solution contained methanol, acetic aid, and double-distilled water in a volumetric ratio of 3:1:6. The gel was then quantified using Gel-Pro Analyzer 4.0 software [50], the intensity of certain protein bands was significantly up- or downregulated based on the gel electrophoresis; these bands were identified and cut out of the gel for in-gel tryptic digestion as previously described [51]. The protein bands were destained in 100 mM NH_4_HCO_3_ containing 30% ACN, then removed supernatant after CCB was completely washed off. The gel slices were vacuum freeze-dried for 30 min. The gel-bound proteins were reduced with 10 mM DTT in 90 μL of 100 mM NH_4_HCO_3_ at 56°C for 30 min, alkylated in 30 mM iodoacetamide in the dark for 20 min, washed with 100 mM NH_4_HCO_3_ for 15 min, dehydrated again with ACN, and finally vacuum freeze-dried. The dry gel pieces were rehydrated with 5 μL of 5 ng of trypsin. Subsequently, 25 μL of 50 mM NH_4_HCO_3_ without trypsin was added after the protein bands were completely swollen at 4°C. Protein digestion was performed overnight at 37°C. The peptides were extracted thrice with 100 μL of 0.1% formic acid and 60% ACN by ultrasonication for 15 min. The isolated peptides were then freeze-dried and stored at -80°C.

### Nano-LC-MS/MS analysis

MS analysis was performed on a LTQ XL mass spectrometer (ThermoFisher, San Jose, CA, USA) coupled with a homemade nano-ionization source, two Finnigan quaternary pumps (a sample pump and an MS pump; LC Packings, San Jose, CA), and an autosampler (LC Packings, San Jose, CA) equipped with a two-position, 10-port valve with a 25 μL sample loop. The 10 ports contained two parallel enrichment columns (Zorbax) that were used to trap the peptides.

Gel bands were identified by 1D-LC-MS/MS, wherein the peptides were only separated by an RP column (BioBasic-C18). Samples were re-dissolved in double-distilled water containing 0.1% FA. All peptides were injected into the sample loop, loaded into the sample pump of an enrichment column, and enriched for 10 min with the mobile phase directed to a waste bottle (the enrichment column contained 100% double-distilled water). Simultaneously, the mobile phase was passed through another enrichment column and directed to the RP column for separation and MS detection by MS pump. The functions of these two enrichment columns were switched by a 10-port valve when peptides were completely enriched after 10 min. The enriched peptides were eluted by the mobile phase from the enrichment column, further separated by the RP column, and detected by MS within the succeeding 80 min. For the mobile phase that was pumped by the MS pump for MS detection, the elution buffer A was 0.1% FA in double-distilled water, whereas the elution buffer B was 0.1% FA in ACN. We used 98% buffer A and 2% buffer B in the first 10 min to equilibrate the RP column, followed by a gradient of 40 min in 40% buffer B, a gradient of 30 min in 90% buffer B, and a gradient of 10 min with 98% buffer A and 2% buffer B to re-equilibrate the columns at a constant flow rate of 150 μL/min. By contrast, the mobile phase was pumped by a sample pump at 100 μL/min.

The 2D-LC-MS/MS analysis was performed as previously described [52] with the same settings and similar principles. The peptides were first separated by an SCX column (BioBasic SCX) with 10 concentration steps of ammonium chloride (dissolved in a specified buffer containing 4% ACN and 0.1% FA), namely, 0, 25, 50, 100, 150, 200, 250, 300, 500, and 1000 mM. The peptides were eluted using different salt steps and further separated by an RP column. For proteomic analysis, 20 μL of the redissolved digested peptides (50 μg samples) were loaded with a flow rate of 150 μL/min for 30 min. Peptides in the gradient elution were trapped and desalted using the exchanged enrichment columns, followed by secondary separation on the RP column. The eluting peptides were introduced to MS online. As described above, the elution buffer A was 0.1% FA in double-distilled water, whereas the elution buffer B was 0.1% FA in ACN. For the mobile phase pumped by the MS pump for MS detection, we used 98% buffer A and 2% buffer B in the first 5 min, followed by a gradient of 45 min in 40% buffer B, a gradient of 30 min in 90% B, and then 20 min in 98% buffer A and 2% buffer B to re-equilibrate the columns at a constant flow rate of 150 μL/min. By contrast, the mobile phase pumped by the sample pump was at 100 μL/min with a constant composition of 98% buffer A and 2% buffer B.

The LTQ mass spectrometer was operated using the “instrument method” of the Xcaliber software (Thermo Finnigan). The electrospray voltage was set to 2.0 kV, whereas the capillary voltage was 4.5 kV. The temperature of the heated capillary was set to 180°C. The data collected during the MS experiment used a full MS scan range of 300 *m/z* to 2000 *m/z*, followed by MS/MS analysis in the positive mode and intervening MS/MS scans on the 10 most intense ions in the MS scan. The collision-induced dissociation had a normalized collision energy of 35%. Parent ions were selected by dynamic exclusion with a repeat count of two, a repeat duration of 30 s, and an exclusive duration of 90 s.

### Data analysis

For 2D-LC-MS/MS, the 10 raw files were searched against an in-house protein database, which was downloaded from the National Center of Biotechnology Information (NCBI, update May 12, 2012; http://www.ncbi.nlm.nih.gov/) and contained 8344 entries on *S. spinosa*. Most of the entries were proteins of *S. spinosa* NRRL 18395, whose genome was sequenced in March 2011 [5]. So the identification of the proteins which were highly conserved in both strains and will give more model regulatory proteins information in *S. spinosa*. Database searches depended on the SEQUEST search engine (a part of the Proteome Discoverer 1.1 software package). The following search parameters were used: only the b and y fragment ions were considered, and two missed cleavages were allowed. Furthermore, the mass tolerances of the precursor and fragmentation ions were 1.5 and 1.0 Da, respectively. The allowable modifications were of cysteine carbamidomethyl as the static modification and methionine oxidation the as variable modification. A target-decoy database from the original (target) and reversed (decoy) databases was established to satisfy a false discovery rate less than 1% for all searches. A protein was considered “identified” when it contained more than two unique peptides or had one unique peptide with no less than seven amino acids and at least three consecutive b- or y-ions. For 1D-LC-MS/MS data analysis, the searches were conducted only with the SEQUEST search engine under the same parameters. Proteins were classified into different functional categories according to the KEGG pathway (http://www.genome.jp/kegg/) and Gene Ontology (http://www.geneontology.org/) databases.

### Semi-quantification analysis of different protein expression

A modified method was used to estimate the changes in protein abundance during fermentation. PAI provides a semi-quantitative measure of protein abundance [16] and represents the number of peptides identified divided by the number of theoretically observable tryptic peptides. For the identified peptides, SEQUEST counted the number of peptide ions with replicates (not the number of unique peptides) of a protein and the number of identified peptides equal to the average value from two replicates. A strict selection of quantified proteins was used in this study, thus, the proteins which had two unique peptides in each MS analysis and have detected in both biological replicates were then quantified and further analyzed for their expression abundance. Theoretically observable peptides were calculated by an online tool, IPEP (http://ipep.moffitt.org/), subjected to trypsin digestion, and detected under electrospray ionization using an ion trap model with two missing cleavages [53].

A total of 689 specific proteins from four time phases were quantified in this study (Figure 2A; Additional file 1: Dataset S1). However, the observed PAI values varied with the total proteins observed in each experiment. The experiments were conducted according to standard operating procedures to compare the PAI values from one time phase to another. When the time phases were comparable, the ratios of the total PAI per protein between replicate time phases had to be similar to each other. Thus, the individual PAI values for each protein from the four time phases were added to determine the ΣPAI_BR1_ and ΣPAI_BR2_ for each of the 689 proteins that were respectively quantified in replicates 1 and 2 (Additional file 3: Table S1). When a protein was not detected in the said time phase, its PAI was 0 and marked with ND. To test whether the summed PAI values of each protein were comparable among time phases, the per protein ratio ΣPAI_BR1/BR2_ was calculated for ΣPAI_BR1_ compared with ΣPAI_BR2_. The average ratio from ΣPAI_BR1/BR2_ was 1.11 ± 0.26, which indicates that a comparison of PAI values between replicates is reasonable. The overall accordance between the total PAI per protein from the replicates was further demonstrated by regression plot of ΣPAI_BR1_ to ΣPAI_BR2_ (Additional file 2: Figure S1), where *R*^2^ was 0.97. Therefore, the two replicates were highly correlated. We were more interested in proteins that were highly expressed in *S. spinosa* proteome in case of such proteins were mostly potential regulatory proteins related to spinosad production. So, we ruled that a protein with a total PAI value greater than three was abundant in *S. spinosa* proteome (Additional file 3: Table S2). Protein abundance was considered significantly up- or downregulated when the PAI ratios between any two time phases were higher than 1.5 or lower than 0.75, respectively.

### Quantitative real-time PCR

Quantitative real-time PCR (qRT-PCR) amplification, detection, and analysis were performed with an ABI 7500 Real-Time PCR System (Applied Biosystems, USA) using Power SYBR® Green PCR Master Mix (Applied Biosystems), as previously described [54]. The transcriptional levels of five proteins involved in different metabolic processes were selected and analyzed by qRT-PCR (MHSM, GS, CNDP, PSDA and probable O-methyltransferase). The sequences of the primers used in real-time PCR were developed with Primer version 5.00 (Premier Biosoft International, Palo Alto, CA) and listed in Additional file 3: Table S4. Transcript generated from the 16S rRNA gene was used for normalization [55].

### Western blot validation

The validation experiments of the 2D-LC-MS/MS results showed the relative abundance of three selected proteins that were differentially expressed in the four time phases. These proteins, namely, MHSM, GS, and CNDP were analyzed using Western blot. Corresponding genes were successfully cloned from the chromosomal DNA according to the published genome sequence of *S. spinosa* NRRL 18395 with three pairs of primers designed by Primer version 5.00 (Premier Biosoft International, Palo Alto, CA) in Additional file 3: Table S4. The three gene fragments were cloned into the pET28a vector according to the manufacturer’s instructions. The resultant plasmid was completely sequenced (Invitrogen, Shanghai, China). Heterologous expression of MHSM, GS, and CNDP in *E. coli* BL21 was performed. The induced MHSM, GS, and CNDP proteins were fused with His_6_-tag and purified by an ÄKTA purifier 100 system (GE Healthcare, USA) according to the protocol of a HisTrap FFcrude 1 mL column (GE Healthcare, USA). The enzyme was then used for anti-serum production in rabbits according to the method of Huang et al. [52]. Proteins from the four phases in *S. spinosa* were extracted according to the aforementioned method. After protein quantization using a 2-D Quant Kit, 20 μg of proteins from each phase was separated by NuPAGE® 4% to 12% Bis-Tris Gel (Invitrogen, USA), and then blotted onto polyvinylidene difluoride (PVDF) membranes (Sigma) using a tank blot apparatus (Toyo, Tokyo, Japan). The transfer, blocking, hybridization, and washing steps were performed according to Huang et al. [52]. The PVDF membranes were incubated with primary rabbit antisera at a dilution of 1:800 and then labeled with anti-rabbit secondary antibody conjugated to goat DyLight 680 (Invitrogen, USA) at a dilution of 1:10 000. The binding of the secondary antibody was detected using the Odyssey infrared imaging system (Li-COR Biosciences, Lincoln, NE).

## Abbreviations

PAI: Protein abundance index; SCX: Strong cation exchange; LTQ: Linear trap quadrupole; NCBI: Center of biotechnology information; IPEP: In silico examination of proteolytic peptides for mass spectrometry; MW: Molecular weight; MHSM: 5-methyltetrahydropteroyltriglutamate-homocysteine S-methyltransferase; GS: Glutamine synthetase; CNDP: Cyclic nucleotide-binding domain-containing protein; PSDA: Polysaccharide deacetylase; qRT-PCR: Quantitative RT-PCR.

## Competing interests

The authors declare that they have no competing interests.

## Authors’ contributions

QY conceived and designed the study, carried out the proteomic studies, qRT-PCR analysis, and drafted the manuscript. XD and LX participated in its design and coordination and helped to draft the manuscript. LX and SL contributed to the proteomic data analysis. YS, ZY and SH participated in western blotting analysis. JR, HH and HH contributed to the bacteria cultures experiments. All authors read and approved the final manuscript.

## Supplementary Material

Additional file 1 **Supplementary datasets, Dataset S1.** Relative quantification analysis of proteins identified from each time phases and biological replicates, respectively. **Dataset S2.** Identification of *S. spinosa* proteins from each time phases and biological replicates. Click here for file

Additional file 2 **Supplementary figures, Figure S1.** Regression plot of the total PAI per protein from the replicates. The overall accordance between the total PAI per protein from the replicates was further demonstrated by regression plots of ΣPAI_BR1_ to ΣPAI_BR2_, where R2 was 0.97. **Figure S2.** Molecular mass and theoretical pI distribution of the total proteome. **Figure S3.** The results of SDS-PAGE were analyzed with Gel-Pro Analyzer 4.0 software. The alterations in protein level were illustrated by a vertical bar chart. The intensity analysis was carried out using GelPro Software (Gel-Pro Analyzer 4.0; Media Cybernitics) and the expression levels were measured as value of IOD (integral optical density) of each bands owned the same MW. Click here for file

Additional file 3 **Supplementary tables, Table S1.** The ratios of the total PAI per protein between replicate time phases. The individual PAI values for each protein from the four time phases were added to determine the ΣPAIBR_1_ and ΣPAIBR_2_ for each of the 689 proteins that were quantified in replicates 1 and 2, respectively. ND, The protein is not detected. **Table S2.** The most abundant proteins in *S. spinosa* (PAI > 3). Averaged PAI ratios from two biological replicates. T2/T1, T3/T1, T4/T1, T3/T2, T4/T2, and T4/T3 represent the six pairs of comparisons of the average PAI for each protein. ND, The protein is not detected. **Table S3.** Proteins for spinosad biosynthesis in *S. spinosa.* The functions of each protein are classified according to their annotated functions in GenBank as well as based on their homology or function, as described in their gene ontology, conserved domains, and KEGG pathways. Genes have been studied and identified to code corresponding proteins in other researches. Total number of identified peptides from two biological replicates were gave. ND, The protein is not detected. **Table S4.** Oligonucleotide primers used in this study. **Table S5.** Enzyme name abbreviations used in this study. Click here for file
