# Supplementary figures and images for: Differential proteomic profiling reveals regulatory proteins and novel links between primary metabolism and spinosad production in Saccharopolyspora spinosa
Source: Microb Cell Fact. 2014 Feb 21;13:27. doi: 10.1186/1475-2859-13-27 (PMC3936707; doi:10.1186/1475-2859-13-27)

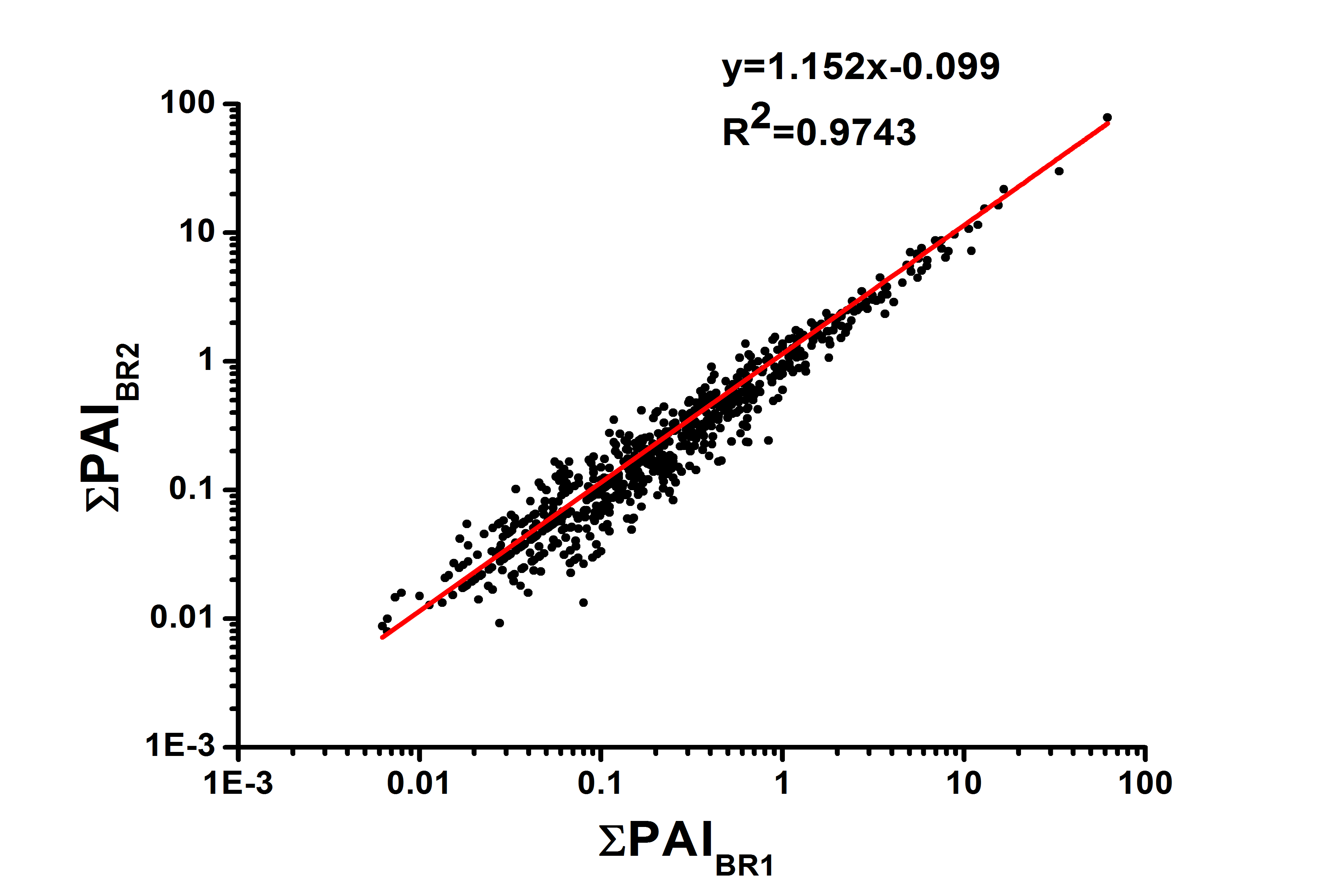

Supplement: Additional file 2 — Supplementary figures, Figure S1. Regression plot of the total PAI per protein from the replicates. The overall accordance between the total PAI per protein from the replicates was further demonstrated by regression plots of ΣPAIBR1 to ΣPAIBR2, where R2 was 0.97. Figure S2. Molecular mass and theoretical pI distribution of the total proteome. Figure S3. The results of SDS-PAGE were analyzed with Gel-Pro Analyzer 4.0 software. The alterations in protein level were illustrated by a vertical bar chart. The intensity analysis was carried out using GelPro Software (Gel-Pro Analyzer 4.0; Media Cybernitics) and the expression levels were measured as value of IOD (integral optical density) of each bands owned the same MW. [file 1475-2859-13-27-S2.zip › 1333445269109951_add4.tiff]

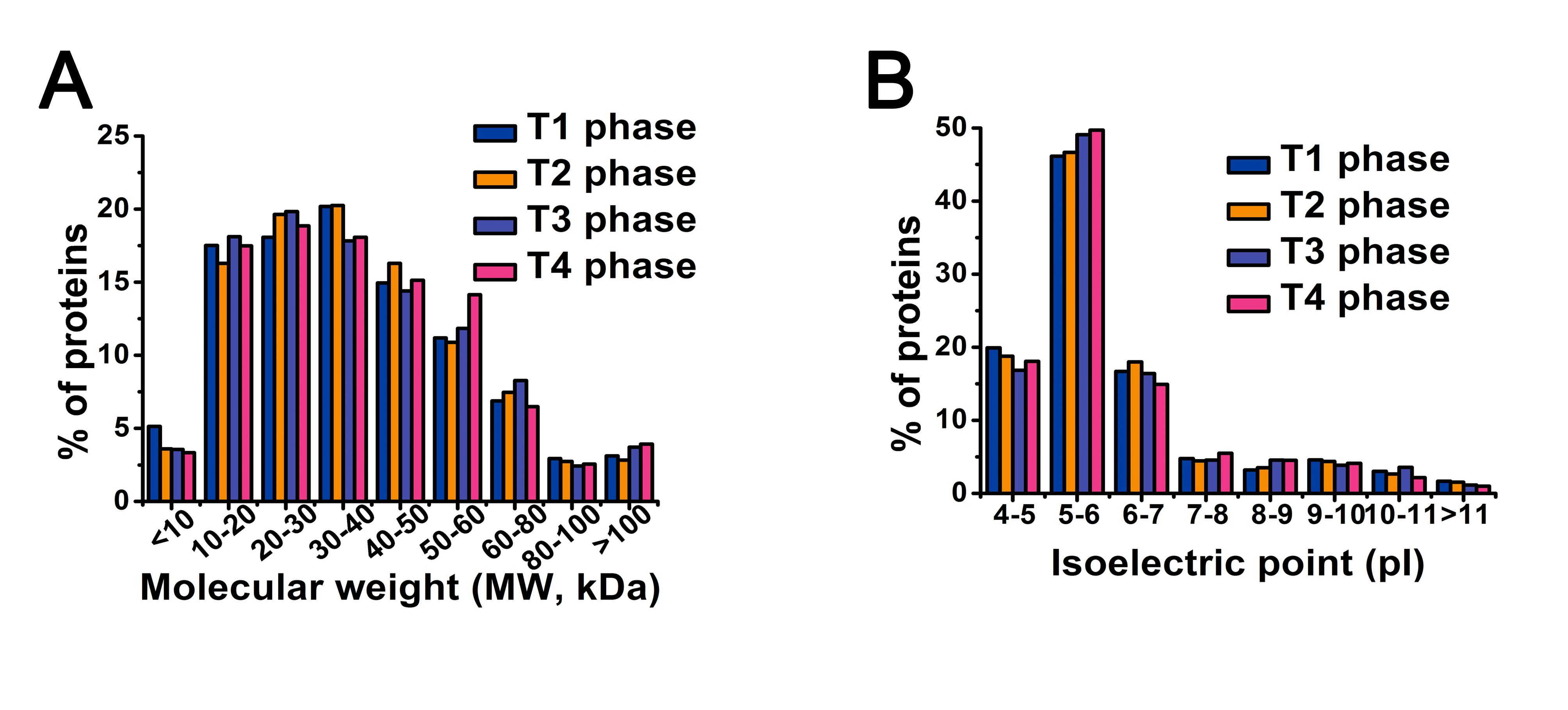

Supplement: Additional file 2 — Supplementary figures, Figure S1. Regression plot of the total PAI per protein from the replicates. The overall accordance between the total PAI per protein from the replicates was further demonstrated by regression plots of ΣPAIBR1 to ΣPAIBR2, where R2 was 0.97. Figure S2. Molecular mass and theoretical pI distribution of the total proteome. Figure S3. The results of SDS-PAGE were analyzed with Gel-Pro Analyzer 4.0 software. The alterations in protein level were illustrated by a vertical bar chart. The intensity analysis was carried out using GelPro Software (Gel-Pro Analyzer 4.0; Media Cybernitics) and the expression levels were measured as value of IOD (integral optical density) of each bands owned the same MW. [file 1475-2859-13-27-S2.zip › 1333445269109951_add5.tiff]

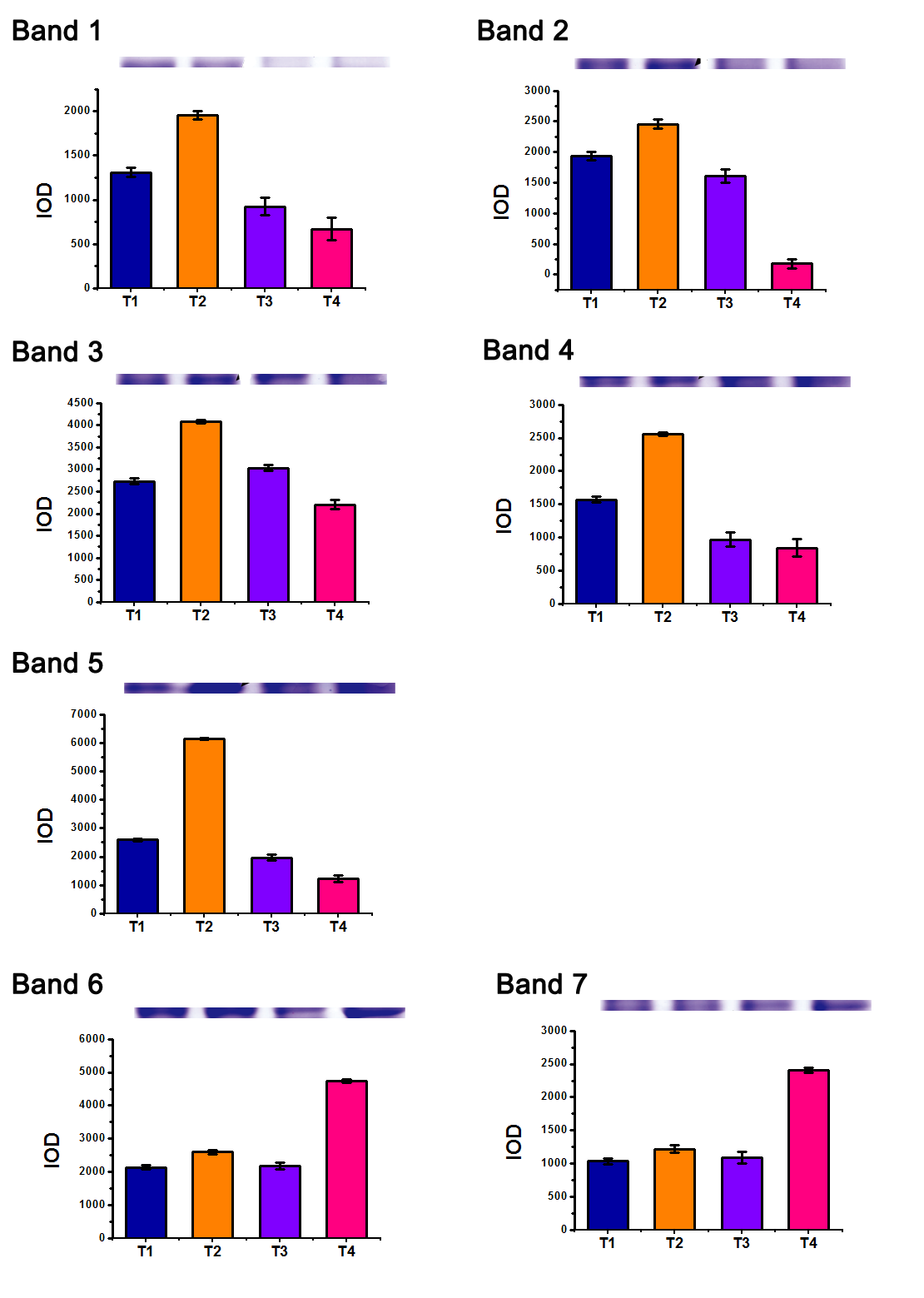

Supplement: Additional file 2 — Supplementary figures, Figure S1. Regression plot of the total PAI per protein from the replicates. The overall accordance between the total PAI per protein from the replicates was further demonstrated by regression plots of ΣPAIBR1 to ΣPAIBR2, where R2 was 0.97. Figure S2. Molecular mass and theoretical pI distribution of the total proteome. Figure S3. The results of SDS-PAGE were analyzed with Gel-Pro Analyzer 4.0 software. The alterations in protein level were illustrated by a vertical bar chart. The intensity analysis was carried out using GelPro Software (Gel-Pro Analyzer 4.0; Media Cybernitics) and the expression levels were measured as value of IOD (integral optical density) of each bands owned the same MW. [file 1475-2859-13-27-S2.zip › 1333445269109951_add6.tiff]
