# Supplementary material for: Differential proteomic profiling reveals regulatory proteins and novel links between primary metabolism and spinosad production in Saccharopolyspora spinosa
Source: Microb Cell Fact. 2014 Feb 21;13:27. doi: 10.1186/1475-2859-13-27 (PMC3936707; doi:10.1186/1475-2859-13-27)
Supplement: Additional file 3 — Supplementary tables, Table S1. The ratios of the total PAI per protein between replicate time phases. The individual PAI values for each protein from the four time phases were added to determine the ΣPAIBR1 and ΣPAIBR2 for each of the 689 proteins that were quantified in replicates 1 and 2, respectively. ND, The protein is not detected. Table S2. The most abundant proteins in S. spinosa (PAI > 3). Averaged PAI ratios from two biological replicates. T2/T1, T3/T1, T4/T1, T3/T2, T4/T2, and T4/T3 represent the six pairs of comparisons of the average PAI for each protein. ND, The protein is not detected. Table S3. Proteins for spinosad biosynthesis in S. spinosa. The functions of each protein are classified according to their annotated functions in GenBank as well as based on their homology or function, as described in their gene ontology, conserved domains, and KEGG pathways. Genes have been studied and identified to code corresponding proteins in other researches. Total number of identified peptides from two biological replicates were gave. ND, The protein is not detected. Table S4. Oligonucleotide primers used in this study. Table S5. Enzyme name abbreviations used in this study. [file 1475-2859-13-27-S3.pdf]

Additional file 1:

Supplementary tables

Table S1 The ratios of the total PAI per protein between replicate time phases

NCBI Accession	PAI value in biological replicate 1				ΣPAI$_{BR1}$[a]	PAI value in biological replicate 2				ΣPAI$_{BR2}$[b]	Ratio ΣPAI$_{BR1/BR2}$
Time phases	T1	T2	T3	T4		T1	T2	T3	T4		
gi348173102	3.86	3.83	0.82	0.34	8.85	4.35	4.20	0.73	0.40	9.68	0.91
gi348173178	10.78	8.70	5.56	8.44	33.48	8.70	10.22	4.52	6.59	30.04	1.11
gi348176726	10.43	41.26	7.04	3.09	61.83	11.09	56.09	8.26	3.35	78.78	0.78
gi348176428	1.74	2.51	0.77	0.58	5.60	1.86	2.71	1.03	0.68	6.27	0.89
gi348174902	3.00	5.17	2.98	0.81	11.95	2.83	5.79	1.95	0.93	11.50	1.04
gi348170321	0.78	1.36	0.62	0.40	3.16	0.69	1.42	0.47	0.44	3.02	1.05
gi348173691	8.75	4.67	2.42	0.75	16.58	8.50	11.00	1.83	0.50	21.83	0.76
gi348169092	1.20	1.08	0.23	0.18	2.69	1.30	0.90	0.20	0.24	2.64	1.02
gi348173083	2.68	3.58	0.74	0.52	7.52	2.32	5.00	0.90	0.52	8.74	0.86
gi348170335	3.81	3.05	0.48	0.19	7.52	4.00	2.76	0.52	0.24	7.52	1.00
gi348175327	2.57	3.70	4.10	5.10	15.47	3.03	3.63	5.07	4.60	16.33	0.95
gi348176809	2.88	0.88	ND	ND	3.77	2.58	0.73	ND	ND	3.31	1.14
gi348169183	3.95	0.74	0.37	ND	5.05	5.53	0.95	0.58	ND	7.05	0.72
gi348169918	4.38	2.50	0.69	0.63	8.19	4.06	2.06	0.81	0.25	7.19	1.14
gi348171102	0.63	0.51	0.23	0.08	1.45	0.31	0.73	0.21	0.06	1.32	1.09
gi348176429	0.46	0.40	0.03	0.01	0.91	0.34	0.52	0.03	0.01	0.91	1.00
gi348170197	0.47	0.39	0.15	0.08	1.09	0.32	0.40	0.18	0.06	0.97	1.13
gi348173100	3.94	1.41	0.18	ND	5.53	3.41	3.06	0.12	ND	6.59	0.84

gi348174397	5.15	4.08	1.23	0.15	10.62	5.69	3.77	1.08	0.15	10.69	0.99
gi348176887	0.81	1.01	0.75	0.37	2.94	0.83	0.80	0.70	0.24	2.57	1.15
gi348171511	0.75	3.15	1.21	0.73	5.85	0.86	2.67	0.68	0.87	5.08	1.15
gi348176808	0.52	0.51	0.11	ND	1.13	0.57	0.50	0.11	ND	1.19	0.95
gi348173909	2.63	0.67	ND	ND	3.29	2.00	0.96	ND	ND	2.96	1.11
gi348173006	0.26	0.17	ND	ND	0.42	0.29	0.24	ND	ND	0.53	0.80
gi348171614	0.34	0.41	0.36	0.20	1.32	0.36	0.37	0.21	0.18	1.12	1.18
gi348175422	1.00	1.48	0.24	0.07	2.79	0.91	1.38	0.29	0.14	2.72	1.03
gi348176443	2.24	3.20	0.76	0.76	6.96	2.24	4.96	1.00	0.52	8.72	0.80
gi348174563	0.64	1.51	0.51	0.08	2.74	0.87	2.14	0.47	0.02	3.51	0.78
gi348176721	5.60	7.40	ND	ND	13.00	4.80	10.60	ND	ND	15.40	0.84
gi348174019	0.81	0.68	0.19	0.12	1.80	0.78	0.96	0.23	0.17	2.14	0.84
gi348170794	0.53	0.36	0.05	ND	0.94	0.53	0.57	0.12	ND	1.23	0.77
gi41350153	0.46	0.73	0.64	0.57	2.40	0.42	0.71	0.47	0.49	2.08	1.15
gi348169997	1.21	1.12	0.23	ND	2.56	1.12	1.21	0.21	ND	2.53	1.01
gi348171612	1.70	1.00	0.20	ND	2.90	1.63	1.00	0.27	ND	2.90	1.00
gi348174564	0.57	0.94	0.32	0.02	1.86	0.53	1.29	0.33	0.02	2.17	0.86
gi348174411	0.71	0.68	0.19	0.20	1.78	0.74	0.52	0.19	0.26	1.71	1.04
gi348176909	0.74	0.60	ND	ND	1.34	0.74	0.20	ND	ND	0.94	1.43
gi348168964	0.18	0.19	0.07	0.01	0.45	0.17	0.18	0.05	0.03	0.44	1.02
gi348172298	0.36	0.73	0.10	0.02	1.20	0.47	0.50	0.06	0.04	1.07	1.12
gi348173515	0.68	0.42	ND	ND	1.11	0.45	0.42	ND	ND	0.88	1.26
gi348173142	1.29	2.17	ND	ND	3.46	1.69	2.80	ND	ND	4.49	0.77
gi348173305	0.67	0.84	0.41	0.05	1.97	0.75	0.66	0.44	0.09	1.94	1.02
gi348174826	0.55	1.38	1.21	1.88	5.01	0.66	1.31	1.90	1.65	5.52	0.91

gi348169301	0.59	0.45	0.17	ND	1.22	0.49	0.51	0.12	ND	1.12	1.09
gi348176933	0.37	0.08	0.04	ND	0.50	0.40	0.09	0.03	ND	0.52	0.95
gi348172504	0.68	1.02	0.14	ND	1.83	0.66	0.63	0.07	ND	1.36	1.35
gi348170152	1.29	1.94	0.26	ND	3.48	0.97	1.97	0.10	ND	3.03	1.15
gi348175715	0.68	1.00	0.09	ND	1.77	0.79	0.84	0.09	ND	1.71	1.03
gi348174864	2.64	1.64	0.86	0.36	5.50	2.21	2.71	1.29	0.64	6.86	0.80
gi348174687	0.38	1.27	0.54	0.08	2.27	0.48	1.29	0.62	0.10	2.49	0.91
gi348172972	0.90	0.71	0.32	0.20	2.12	0.93	1.07	0.20	0.17	2.37	0.90
gi348175040	0.71	0.71	0.31	0.18	1.90	0.69	0.63	0.25	0.18	1.75	1.09
gi348171742	0.40	0.25	0.13	ND	0.78	0.46	0.31	0.09	ND	0.86	0.91
gi348176468	1.26	1.22	0.33	ND	2.81	1.56	0.81	0.33	ND	2.70	1.04
gi290543607	0.68	ND	0.04	ND	0.72	0.54	ND	0.04	ND	0.58	1.24
gi348176851	1.89	1.83	0.83	0.28	4.83	1.39	3.00	1.00	0.22	5.61	0.86
gi348174074	0.79	2.95	2.65	1.53	7.93	0.60	2.81	1.79	1.21	6.42	1.24
gi348176475	0.70	0.34	0.34	0.09	1.47	0.74	0.43	0.17	0.17	1.51	0.97
gi348176895	0.49	0.31	0.21	0.06	1.07	0.57	0.45	0.10	0.04	1.16	0.92
gi348169779	0.45	0.70	0.10	ND	1.25	0.45	0.63	0.12	ND	1.21	1.03
gi348170208	0.79	0.93	1.10	0.86	3.67	0.71	1.14	0.90	1.00	3.76	0.97
gi348170176	0.85	0.69	0.10	ND	1.64	0.62	0.56	0.38	ND	1.56	1.05
gi348169193	0.56	0.37	0.04	0.12	1.09	0.60	0.74	0.07	0.09	1.49	0.73
gi348171262	0.62	0.69	0.42	0.38	2.12	0.71	0.83	0.46	0.25	2.25	0.94
gi348171433	0.25	0.11	0.13	0.17	0.65	0.25	0.15	0.10	0.08	0.57	1.14
gi348170186	0.25	0.33	0.02	ND	0.60	0.23	0.32	0.03	ND	0.58	1.03
gi348173300	0.66	0.73	0.09	ND	1.48	0.61	0.77	0.07	ND	1.45	1.02
gi348176452	0.11	0.13	0.05	0.03	0.33	0.08	0.06	0.02	0.05	0.22	1.49

gi348173307	0.65	1.12	0.67	0.14	2.58	0.67	1.09	0.67	0.05	2.49	1.04
gi348174969	0.45	0.32	ND	ND	0.77	0.42	0.40	ND	ND	0.82	0.94
gi348171500	0.36	0.78	0.34	0.26	1.74	0.42	0.95	0.50	0.50	2.36	0.74
gi348174688	0.54	1.10	0.42	0.04	2.10	0.48	0.74	0.26	0.04	1.52	1.38
gi348170524	1.50	1.56	ND	0.22	3.28	1.61	1.22	ND	0.17	3.00	1.09
gi348169435	0.77	1.66	ND	ND	2.43	0.46	2.49	ND	ND	2.94	0.83
gi348176451	0.08	0.07	ND	ND	0.15	0.07	0.03	ND	ND	0.11	1.39
gi348176729	0.27	0.27	0.06	0.03	0.64	0.35	0.16	0.04	0.06	0.61	1.05
gi348175423	0.46	0.82	0.29	0.09	1.66	0.30	0.68	0.30	0.20	1.48	1.12
gi348170005	0.22	0.23	0.08	0.03	0.56	0.13	0.29	0.05	0.03	0.50	1.13
gi348175295	0.43	0.48	0.34	0.33	1.59	0.38	0.41	0.57	0.24	1.60	0.99
gi348169090	0.22	0.32	0.12	0.10	0.75	0.29	0.23	0.09	0.06	0.67	1.12
gi348174424	0.29	0.23	ND	ND	0.52	0.27	0.22	ND	ND	0.48	1.08
gi348174689	0.55	1.00	0.09	ND	1.64	0.77	1.14	0.05	ND	1.95	0.84
gi348169412	0.17	0.22	0.13	0.03	0.56	0.17	0.12	0.06	0.05	0.40	1.40
gi348172319	3.43	2.00	0.43	ND	5.86	4.14	2.00	1.43	ND	7.57	0.77
gi348169077	0.13	0.10	ND	ND	0.23	0.07	0.08	ND	ND	0.16	1.47
gi348170709	0.19	0.12	ND	ND	0.31	0.21	0.10	ND	ND	0.30	1.03
gi348176409	0.41	0.34	0.07	ND	0.81	0.49	0.42	0.10	ND	1.02	0.80
gi348171389	1.15	0.35	ND	ND	1.50	1.60	0.30	ND	ND	1.90	0.79
gi348173043	2.09	1.55	1.55	0.36	5.55	1.64	1.55	0.73	0.55	4.45	1.24
gi348171450	0.27	0.46	0.20	0.07	1.00	0.32	0.33	0.18	0.05	0.88	1.14
gi348170177	0.49	0.20	0.04	0.07	0.80	0.49	0.62	0.04	0.04	1.20	0.67
gi348176425	0.37	0.13	0.05	ND	0.55	0.23	0.20	0.07	ND	0.50	1.10
gi348176852	1.17	1.67	2.56	0.89	6.28	1.17	2.11	1.83	1.00	6.11	1.03

gi348171558	1.17	2.28	1.17	0.50	5.11	1.17	1.89	1.50	0.44	5.00	1.02
gi348174006	0.21	0.19	ND	ND	0.40	0.25	0.29	ND	ND	0.55	0.73
gi348169302	0.27	0.32	0.08	ND	0.67	0.21	0.36	0.05	ND	0.63	1.06
gi348176762	0.25	0.18	0.04	0.04	0.51	0.27	0.23	0.05	0.06	0.61	0.83
gi348170205	0.30	0.60	0.21	0.24	1.34	0.13	0.33	0.16	0.21	0.84	1.61
gi348170064	0.51	0.95	1.41	1.24	4.11	0.46	0.62	0.78	1.03	2.89	1.42
gi348176508	0.08	0.09	0.02	ND	0.18	0.11	0.06	0.02	ND	0.19	0.94
gi348169195	0.22	0.23	ND	ND	0.44	0.25	0.10	ND	ND	0.35	1.26
gi348176430	0.31	0.13	0.06	ND	0.50	0.32	0.06	0.08	ND	0.47	1.07
gi348173189	0.33	0.42	ND	ND	0.75	0.35	0.23	ND	ND	0.58	1.30
gi348171651	0.73	0.38	0.12	ND	1.23	0.73	0.65	0.12	ND	1.50	0.82
gi348172112	0.10	0.06	ND	ND	0.16	0.06	0.03	ND	ND	0.09	1.75
gi348171813	0.43	0.38	0.38	0.31	1.50	0.36	0.60	0.43	0.38	1.76	0.85
gi348176722	0.78	1.13	0.17	ND	2.09	0.65	1.30	0.35	ND	2.30	0.91
gi348172782	0.69	1.00	0.12	ND	1.81	0.46	0.92	0.08	ND	1.46	1.24
gi348171928	0.67	0.22	0.30	ND	1.19	0.59	0.93	0.22	ND	1.74	0.68
gi348173521	0.42	0.14	ND	ND	0.56	0.40	0.16	ND	ND	0.56	1.00
gi348169429	0.32	0.39	0.05	0.09	0.84	0.30	0.67	0.07	0.04	1.07	0.79
gi348176397	0.27	0.21	0.05	ND	0.52	0.29	0.10	0.08	ND	0.46	1.14
gi348175373	0.47	1.06	1.25	1.81	4.58	0.47	0.89	0.92	1.81	4.08	1.12
gi348174207	0.13	0.11	0.05	0.02	0.30	0.14	0.11	0.02	0.02	0.29	1.05
gi348176831	0.15	0.04	0.13	0.10	0.42	0.09	0.04	0.13	0.11	0.36	1.18
gi348176412	0.35	0.25	0.04	ND	0.65	0.35	0.33	0.21	ND	0.90	0.72
gi348169513	0.21	0.45	ND	ND	0.66	0.20	0.32	ND	ND	0.51	1.29
gi348176396	0.25	0.17	0.09	0.07	0.58	0.23	0.12	0.03	0.04	0.42	1.38

gi348169208	0.19	0.20	0.07	0.15	0.61	0.12	0.21	0.18	0.16	0.67	0.90
gi348176399	0.29	0.09	ND	ND	0.38	0.24	0.16	ND	ND	0.40	0.95
gi348170217	0.29	0.33	0.22	0.42	1.25	0.25	0.27	0.18	0.49	1.20	1.05
gi348173538	0.19	0.08	ND	0.02	0.30	0.07	0.13	ND	0.02	0.23	1.32
gi348176424	0.52	0.06	ND	ND	0.58	0.42	0.65	ND	ND	1.06	0.55
gi348171298	0.36	0.61	0.50	0.55	2.02	0.23	0.91	0.32	0.73	2.18	0.93
gi348171293	0.21	ND	0.11	0.04	0.36	0.18	ND	0.08	0.03	0.29	1.24
gi348174297	0.23	0.11	0.06	ND	0.39	0.33	0.11	0.09	ND	0.53	0.74
gi348170589	0.15	0.22	0.08	0.04	0.49	0.16	0.25	0.05	0.07	0.53	0.92
gi348176682	0.16	0.41	0.02	0.05	0.64	0.16	0.24	0.03	0.09	0.53	1.20
gi348174426	0.23	0.13	ND	ND	0.37	0.28	0.25	ND	ND	0.53	0.69
gi348172413	0.39	0.06	ND	ND	0.44	0.11	0.06	ND	ND	0.17	2.67
gi348176208	0.34	0.49	0.93	0.73	2.49	0.34	0.78	0.51	0.80	2.44	1.02
gi348174802	0.16	0.10	ND	ND	0.27	0.10	0.05	ND	ND	0.15	1.77
gi348169164	0.37	0.21	0.05	ND	0.63	0.37	0.26	0.08	ND	0.71	0.89
gi348170647	0.17	0.16	0.05	ND	0.38	0.17	0.11	0.06	ND	0.35	1.11
gi348176422	0.37	0.11	0.09	ND	0.57	0.09	0.29	0.11	ND	0.49	1.18
gi348173113	0.11	ND	ND	ND	0.11	0.11	ND	ND	ND	0.11	1.00
gi348174007	0.15	0.06	ND	ND	0.21	0.08	0.06	ND	ND	0.14	1.50
gi348176674	0.20	0.08	ND	ND	0.28	0.23	0.16	ND	ND	0.39	0.72
gi348175334	0.38	0.15	0.09	ND	0.62	0.24	0.50	0.06	ND	0.79	0.78
gi348171442	0.15	0.19	ND	ND	0.34	0.09	0.17	ND	ND	0.27	1.26
gi348174565	0.21	0.13	ND	ND	0.33	0.08	0.06	ND	ND	0.14	2.33
gi348173201	0.16	ND	ND	ND	0.16	0.17	ND	ND	ND	0.17	0.93
gi348174445	0.16	0.69	0.06	ND	0.90	0.16	0.59	0.02	ND	0.77	1.17

gi348170813	0.22	0.10	ND	ND	0.32	0.23	0.05	ND	ND	0.28	1.12
gi348169787	0.15	0.06	ND	ND	0.21	0.09	0.09	ND	ND	0.19	1.13
gi348172545	0.16	0.06	0.05	0.02	0.29	0.13	0.12	0.05	0.02	0.33	0.89
gi348172687	0.16	0.41	0.08	0.05	0.70	0.14	0.39	0.04	0.03	0.59	1.18
gi348177056	0.21	0.14	ND	ND	0.36	0.13	0.16	ND	ND	0.29	1.25
gi348176471	0.16	0.09	0.06	0.05	0.36	0.19	0.05	0.05	0.03	0.32	1.12
gi348176498	0.08	ND	ND	ND	0.08	0.01	ND	ND	ND	0.01	6.00
gi348171330	0.10	0.10	ND	ND	0.19	0.12	0.05	ND	ND	0.17	1.14
gi348171412	0.18	0.58	0.46	0.34	1.57	0.25	0.69	0.48	0.24	1.66	0.95
gi348176769	0.32	0.32	ND	ND	0.63	0.05	0.18	ND	ND	0.24	2.67
gi348173953	0.20	0.29	0.29	0.18	0.95	0.07	0.21	0.04	0.20	0.52	1.83
gi348176597	0.14	0.21	0.21	0.07	0.63	0.14	0.31	0.05	0.10	0.59	1.06
gi348172933	0.10	0.07	0.02	ND	0.19	0.16	0.05	0.03	ND	0.23	0.84
gi348173513	0.73	1.07	ND	ND	1.80	0.53	0.53	ND	ND	1.07	1.69
gi348173198	0.18	0.08	ND	ND	0.26	0.21	0.08	ND	ND	0.30	0.89
gi348175863	0.18	0.70	0.08	ND	0.97	0.15	0.59	0.03	ND	0.77	1.26
gi348175222	0.48	0.65	ND	ND	1.13	0.52	0.74	ND	ND	1.26	0.90
gi348176407	0.22	0.08	ND	ND	0.29	0.18	0.20	ND	ND	0.37	0.79
gi348171814	0.35	0.45	0.26	0.13	1.19	0.48	0.42	0.19	0.23	1.32	0.90
gi348176850	0.23	0.15	0.15	0.11	0.64	0.11	0.11	0.04	0.17	0.43	1.50
gi348172300	0.38	0.21	ND	ND	0.59	0.10	0.17	ND	ND	0.28	2.13
gi348170818	0.73	0.27	ND	ND	1.00	0.40	0.40	ND	ND	0.80	1.25
gi348174635	0.12	0.19	ND	ND	0.31	0.11	0.19	ND	ND	0.29	1.04
gi348170244	0.19	0.20	0.04	0.06	0.48	0.19	0.11	0.07	0.11	0.48	1.00
gi348175322	0.10	0.14	ND	ND	0.24	0.12	0.16	ND	ND	0.29	0.83

gi348169633	0.37	0.26	ND	ND	0.63	0.33	0.44	ND	ND	0.78	0.81
gi348176426	0.15	0.23	0.12	ND	0.51	0.20	0.12	0.06	ND	0.38	1.32
gi348176411	0.21	0.29	0.15	ND	0.65	0.19	0.15	0.10	ND	0.44	1.48
gi348169384	0.37	0.15	ND	ND	0.52	0.37	0.19	ND	ND	0.56	0.93
gi348174790	0.19	0.12	ND	ND	0.31	0.15	0.15	ND	ND	0.31	1.00
gi348171275	0.16	0.03	0.10	ND	0.30	0.26	0.05	0.07	ND	0.38	0.78
gi348172983	0.21	0.15	0.11	ND	0.47	0.15	0.21	0.09	ND	0.45	1.05
gi348169223	1.00	ND	ND	ND	1.00	0.60	ND	ND	ND	0.60	1.67
gi41350159	0.01	0.01	0.01	ND	0.03	0.01	0.01	0.01	ND	0.02	1.69
gi348169040	0.06	0.04	ND	ND	0.10	0.03	0.04	ND	ND	0.06	1.56
gi348176924	0.26	0.32	ND	ND	0.59	0.35	0.47	ND	ND	0.82	0.71
gi348175390	0.24	0.14	0.11	0.16	0.65	0.22	0.08	0.22	0.22	0.73	0.89
gi348176398	0.28	0.13	ND	ND	0.41	0.44	0.28	ND	ND	0.72	0.57
gi348174871	0.09	0.05	0.02	0.02	0.17	0.07	0.07	0.02	0.02	0.17	1.00
gi41350151	0.20	0.20	ND	ND	0.41	0.14	0.09	ND	ND	0.23	1.80
gi348172783	2.25	3.50	3.50	1.75	11.00	2.50	3.00	1.25	0.50	7.25	1.52
gi348172775	0.14	0.35	0.35	0.05	0.89	0.16	0.13	0.16	0.05	0.49	1.81
gi348169763	0.39	0.17	0.09	ND	0.65	0.61	0.22	0.30	ND	1.13	0.58
gi348174004	0.17	0.32	0.15	0.06	0.70	0.21	0.19	0.09	0.06	0.55	1.28
gi348176395	0.13	0.11	ND	0.04	0.28	0.15	0.07	ND	0.03	0.25	1.11
gi348173522	0.11	0.05	ND	ND	0.15	0.07	0.06	ND	ND	0.13	1.18
gi348176419	0.18	0.24	0.12	ND	0.53	0.18	0.12	0.10	ND	0.39	1.35
gi348175109	0.17	ND	ND	ND	0.17	0.17	ND	ND	ND	0.17	1.00
gi348169236	0.04	0.05	ND	0.01	0.10	0.03	0.05	ND	0.01	0.09	1.05
gi348176767	0.12	0.39	ND	ND	0.52	0.26	0.26	ND	ND	0.52	1.00

gi348169091	0.11	0.09	0.04	ND	0.24	0.07	0.09	0.03	ND	0.19	1.29
gi348169576	0.11	0.14	0.10	0.08	0.43	0.11	0.31	0.10	0.06	0.57	0.76
gi348171757	0.24	0.06	0.06	ND	0.35	0.15	0.29	0.15	ND	0.59	0.60
gi348174428	0.06	0.09	ND	ND	0.15	0.09	0.10	ND	ND	0.19	0.83
gi348168969	0.16	0.18	ND	ND	0.33	0.25	0.08	ND	ND	0.33	1.00
gi348176884	0.12	0.22	0.12	ND	0.46	0.09	0.05	0.03	ND	0.17	2.73
gi348176414	0.14	ND	ND	ND	0.14	0.18	ND	ND	ND	0.18	0.80
gi348170434	0.21	0.18	ND	ND	0.39	0.11	0.08	ND	ND	0.18	2.14
gi348176469	0.16	ND	ND	ND	0.16	0.14	ND	ND	ND	0.14	1.14
gi348174014	0.19	0.38	0.10	ND	0.67	0.12	0.45	0.05	ND	0.62	1.08
gi348169562	0.08	0.11	ND	0.02	0.22	0.06	0.09	ND	0.02	0.18	1.24
gi348171376	0.07	0.02	ND	ND	0.09	0.06	0.03	ND	ND	0.09	1.00
gi348171071	0.15	ND	ND	ND	0.15	0.11	ND	ND	ND	0.11	1.33
gi348170477	0.17	0.47	ND	ND	0.64	0.09	0.57	ND	ND	0.66	0.97
gi348174145	0.20	0.05	ND	ND	0.25	0.23	0.10	ND	ND	0.33	0.77
gi348169484	0.10	0.06	ND	ND	0.16	0.08	0.05	ND	ND	0.13	1.30
gi348171447	0.29	0.61	0.25	ND	1.14	0.29	0.46	0.07	ND	0.82	1.39
gi348169042	0.29	0.32	ND	ND	0.61	0.25	0.07	ND	ND	0.32	1.89
gi348176671	0.24	0.18	ND	ND	0.42	0.15	0.24	ND	ND	0.39	1.08
gi13162637	0.02	0.04	ND	ND	0.06	0.03	0.03	ND	ND	0.06	1.06
gi348169479	0.12	0.14	ND	ND	0.25	0.14	0.15	ND	ND	0.29	0.88
gi348172449	0.02	0.02	ND	ND	0.03	0.01	0.03	ND	ND	0.04	0.87
gi348173990	0.13	0.15	ND	ND	0.27	0.11	0.11	ND	ND	0.22	1.25
gi348168991	0.39	1.06	ND	ND	1.44	0.72	1.28	ND	ND	2.00	0.72
gi348171501	0.09	0.25	0.11	0.06	0.51	0.10	0.19	0.06	0.06	0.41	1.24

gi348169336	0.78	1.00	0.44	ND	2.22	0.67	0.56	0.44	ND	1.67	1.33
gi348168944	0.11	ND	ND	ND	0.11	0.05	ND	ND	ND	0.05	2.33
gi348175473	0.09	0.06	ND	0.04	0.19	0.19	0.02	ND	0.05	0.26	0.71
gi348169089	0.09	0.08	0.05	0.04	0.26	0.15	0.12	0.03	0.04	0.33	0.77
gi348170090	0.16	0.16	ND	ND	0.31	0.11	0.09	ND	ND	0.20	1.56
gi348171622	0.70	0.60	ND	ND	1.30	1.20	0.40	ND	ND	1.60	0.81
gi348175425	0.11	0.16	ND	ND	0.28	0.20	0.07	ND	ND	0.26	1.06
gi348169189	0.08	0.07	ND	ND	0.15	0.11	0.06	ND	ND	0.17	0.87
gi348173114	0.16	0.05	ND	ND	0.20	0.32	0.09	ND	ND	0.41	0.50
gi348169079	0.30	ND	ND	ND	0.30	0.48	ND	ND	ND	0.48	0.64
gi348169163	0.12	0.05	ND	ND	0.17	0.08	0.15	ND	ND	0.24	0.71
gi348175267	0.27	0.31	ND	0.15	0.73	0.23	0.08	ND	0.12	0.42	1.73
gi348170206	0.09	0.19	0.18	0.09	0.55	0.11	0.16	0.15	0.12	0.54	1.03
gi348175112	0.18	0.05	0.13	ND	0.37	0.05	0.13	0.08	ND	0.26	1.40
gi348171821	0.06	0.13	0.09	0.04	0.33	0.04	0.17	0.12	0.08	0.42	0.79
gi348175317	0.19	ND	ND	ND	0.19	0.36	ND	ND	ND	0.36	0.54
gi348169294	0.29	0.08	ND	ND	0.38	0.38	0.13	ND	ND	0.50	0.75
gi348176685	0.05	0.05	ND	ND	0.10	0.05	0.05	ND	ND	0.10	1.00
gi348170940	0.08	0.02	ND	ND	0.10	0.12	0.06	ND	ND	0.17	0.60
gi348176725	0.78	1.67	ND	1.22	3.67	0.56	1.33	ND	1.44	3.33	1.10
gi348172925	0.13	0.20	0.15	0.09	0.57	0.17	0.22	0.06	0.04	0.48	1.19
gi348176473	0.19	0.24	0.05	ND	0.49	0.32	0.32	0.05	ND	0.70	0.69
gi348171838	0.18	ND	0.08	ND	0.25	0.33	ND	0.08	ND	0.40	0.63
gi348169354	0.10	0.12	ND	ND	0.22	0.06	0.04	ND	ND	0.10	2.14
gi348170207	0.12	0.17	ND	ND	0.29	0.07	0.15	ND	ND	0.22	1.31

gi348172420	0.22	ND	ND	ND	0.22	0.13	ND	ND	ND	0.13	1.75
gi348169342	0.28	0.60	0.16	0.20	1.24	0.44	0.88	0.24	0.12	1.68	0.74
gi348175004	0.07	0.05	ND	ND	0.12	0.08	0.03	ND	ND	0.11	1.09
gi348169591	0.21	0.09	ND	ND	0.29	0.12	0.18	ND	ND	0.29	1.00
gi348171783	0.21	ND	ND	ND	0.21	0.18	ND	ND	ND	0.18	1.17
gi348169191	0.08	ND	ND	ND	0.08	0.07	ND	ND	ND	0.07	1.17
gi348169207	0.17	ND	ND	ND	0.17	0.10	ND	ND	ND	0.10	1.75
gi348169398	0.16	0.20	ND	ND	0.36	0.07	0.14	ND	ND	0.20	1.78
gi348169469	0.11	0.07	ND	ND	0.18	0.11	0.05	ND	ND	0.16	1.10
gi348173002	0.27	0.54	0.27	0.15	1.23	0.27	0.35	0.12	0.15	0.88	1.39
gi348171396	0.03	ND	ND	ND	0.03	0.02	ND	ND	ND	0.02	1.50
gi348173981	0.19	0.22	ND	ND	0.41	0.38	0.53	ND	ND	0.91	0.45
gi348175288	0.03	ND	ND	ND	0.03	0.02	ND	ND	ND	0.02	1.20
gi348176420	0.05	0.06	ND	ND	0.11	0.06	0.03	ND	ND	0.08	1.30
gi348171791	0.03	ND	ND	ND	0.03	0.01	ND	ND	ND	0.01	3.00
gi348174413	0.08	ND	ND	ND	0.08	0.05	ND	ND	ND	0.05	1.50
gi348174234	0.04	0.03	ND	ND	0.06	0.02	0.01	ND	ND	0.03	2.00
gi348176609	0.08	ND	ND	ND	0.08	0.03	ND	ND	ND	0.03	3.00
gi348171466	0.09	0.18	0.04	ND	0.31	0.10	0.16	0.03	ND	0.29	1.05
gi348173931	0.13	0.15	0.06	ND	0.33	0.21	0.19	0.08	ND	0.48	0.70
gi348176520	0.15	0.22	ND	ND	0.37	0.15	0.17	ND	ND	0.32	1.15
gi348170936	0.06	0.07	0.04	0.04	0.21	0.05	0.08	0.06	0.04	0.23	0.91
gi348172860	0.07	ND	ND	ND	0.07	0.02	ND	ND	ND	0.02	3.00
gi348169396	0.07	0.09	ND	ND	0.15	0.05	0.10	ND	ND	0.15	1.00
gi348175716	0.11	0.19	0.09	ND	0.40	0.25	0.19	0.08	ND	0.51	0.78

gi348170982	0.08	ND	ND	ND	0.08	0.07	ND	ND	ND	0.07	1.20
gi348173992	0.07	ND	ND	ND	0.07	0.07	ND	ND	ND	0.07	1.00
gi348172851	0.24	0.36	0.08	ND	0.68	0.24	0.48	0.24	ND	0.96	0.71
gi348176700	0.10	0.53	0.12	0.12	0.86	0.15	0.10	0.24	0.25	0.75	1.16
gi348172665	0.13	0.28	ND	ND	0.41	0.22	0.24	ND	ND	0.46	0.90
gi348175409	0.06	0.06	ND	ND	0.13	0.04	0.06	ND	ND	0.11	1.20
gi348175436	0.09	0.03	ND	ND	0.12	0.04	0.04	ND	ND	0.09	1.33
gi348175296	0.24	0.40	ND	ND	0.64	0.20	0.16	ND	ND	0.36	1.78
gi348172896	0.14	0.39	0.45	0.30	1.27	0.07	0.11	0.48	0.43	1.09	1.17
gi348171474	0.13	0.28	0.07	ND	0.48	0.09	0.28	0.04	ND	0.41	1.16
gi348172143	0.30	ND	0.20	ND	0.50	0.25	ND	0.15	ND	0.40	1.25
gi348175433	0.18	0.06	ND	ND	0.24	0.09	0.06	ND	ND	0.15	1.60
gi348172914	2.00	1.67	ND	ND	3.67	0.67	1.67	ND	ND	2.33	1.57
gi348175213	0.10	0.09	0.12	ND	0.31	0.10	0.07	0.09	ND	0.26	1.20
gi348169144	0.29	0.29	ND	ND	0.57	0.19	0.33	ND	ND	0.52	1.09
gi348173036	0.29	0.29	0.10	ND	0.67	0.52	0.33	0.24	ND	1.10	0.61
gi348171702	0.06	0.05	0.03	ND	0.14	0.09	0.05	0.03	ND	0.17	0.82
gi348170525	0.67	1.11	0.33	ND	2.11	0.56	0.56	0.78	ND	1.89	1.12
gi347438908	0.08	0.08	ND	ND	0.16	0.05	0.08	ND	ND	0.13	1.25
gi348171942	0.05	0.11	0.02	0.02	0.20	0.05	0.02	0.02	0.05	0.15	1.36
gi348170580	0.05	0.02	ND	ND	0.07	0.03	0.03	ND	ND	0.06	1.17
gi348171266	0.07	0.04	ND	ND	0.11	0.08	0.04	ND	ND	0.12	0.89
gi348171970	0.04	0.05	ND	ND	0.09	0.02	0.05	ND	ND	0.07	1.38
gi348170939	0.03	0.12	0.11	0.18	0.44	0.04	0.07	0.08	0.19	0.38	1.19
gi348173072	0.16	0.19	ND	0.06	0.41	0.13	0.06	ND	0.13	0.31	1.30

gi348176594	0.03	0.03	ND	ND	0.06	0.04	0.02	ND	ND	0.06	1.00
gi348170795	0.09	0.03	ND	ND	0.12	0.14	0.09	ND	ND	0.22	0.54
gi348170779	0.09	0.11	ND	ND	0.21	0.11	0.09	ND	ND	0.21	1.00
gi348170871	0.31	0.25	ND	0.13	0.69	0.13	0.25	ND	0.13	0.50	1.38
gi348176863	0.06	0.13	ND	0.03	0.22	0.03	0.05	ND	0.03	0.12	1.90
gi348173054	0.04	0.02	ND	ND	0.06	0.04	0.01	ND	ND	0.06	1.00
gi348176766	0.10	0.15	ND	0.06	0.31	0.10	0.13	ND	0.17	0.40	0.79
gi348174892	0.06	0.07	0.09	ND	0.22	0.07	0.04	0.02	ND	0.14	1.64
gi348169159	0.08	ND	ND	ND	0.08	0.05	ND	ND	ND	0.05	1.67
gi348171923	0.05	0.05	ND	ND	0.09	0.03	0.05	ND	ND	0.08	1.25
gi13162644	0.08	0.23	ND	ND	0.31	0.10	0.11	ND	ND	0.21	1.46
gi348172910	0.04	ND	ND	ND	0.04	0.05	ND	ND	ND	0.05	0.83
gi348174886	0.11	0.18	ND	ND	0.30	0.14	0.16	ND	ND	0.30	1.00
gi348169291	0.11	ND	ND	ND	0.11	0.07	ND	ND	ND	0.07	1.67
gi348173101	0.08	ND	ND	ND	0.08	0.05	ND	ND	ND	0.05	1.67
gi348175718	0.63	0.38	ND	ND	1.00	1.00	0.38	ND	ND	1.38	0.73
gi348176800	0.08	0.06	ND	ND	0.15	0.05	0.03	ND	ND	0.08	1.80
gi348171620	0.14	0.11	ND	ND	0.25	0.11	0.06	ND	ND	0.17	1.50
gi348172883	0.07	ND	ND	ND	0.07	0.03	ND	ND	ND	0.03	2.50
gi348169928	0.45	1.64	0.64	0.82	3.55	0.55	0.64	1.00	1.09	3.27	1.08
gi348173304	0.05	0.04	0.03	ND	0.12	0.03	0.02	0.04	ND	0.09	1.33
gi348172785	0.08	ND	ND	ND	0.08	0.06	ND	ND	ND	0.06	1.25
gi348172416	0.71	ND	ND	ND	0.71	0.86	ND	ND	ND	0.86	0.83
gi348173138	0.06	0.02	ND	ND	0.08	0.05	0.02	ND	ND	0.07	1.17
gi348174973	0.16	0.06	ND	ND	0.22	0.13	0.06	ND	ND	0.19	1.17

gi348176849	0.10	0.08	ND	ND	0.18	0.10	0.06	ND	ND	0.16	1.13
gi348176666	0.05	0.10	ND	ND	0.15	0.09	0.04	ND	ND	0.13	1.17
gi348176415	0.14	0.11	ND	ND	0.26	0.06	0.06	ND	ND	0.11	2.25
gi348176418	0.18	0.07	ND	ND	0.25	0.11	0.07	ND	ND	0.18	1.40
gi348171407	0.03	0.02	ND	ND	0.05	0.04	0.02	ND	ND	0.06	0.89
gi348174787	0.63	ND	ND	ND	0.63	1.38	ND	ND	ND	1.38	0.45
gi348169583	0.17	0.14	0.17	0.17	0.66	0.21	0.14	0.10	0.14	0.59	1.12
gi348176819	0.09	ND	ND	ND	0.09	0.04	ND	ND	ND	0.04	2.50
gi348169249	0.22	ND	ND	ND	0.22	0.26	ND	ND	ND	0.26	0.83
gi348171617	0.11	ND	ND	ND	0.11	0.11	ND	ND	ND	0.11	1.00
gi348174232	0.31	0.31	ND	ND	0.63	0.19	0.31	ND	ND	0.50	1.25
gi348171859	0.01	0.02	ND	ND	0.03	0.02	0.02	ND	ND	0.03	0.83
gi348169200	0.08	0.08	ND	ND	0.17	0.10	0.10	ND	ND	0.21	0.80
gi348176626	0.07	ND	ND	ND	0.07	0.04	ND	ND	ND	0.04	2.00
gi348171335	0.04	0.02	ND	ND	0.05	0.04	0.03	ND	ND	0.06	0.86
gi348172905	0.05	0.16	0.06	0.06	0.33	0.08	0.13	0.07	0.04	0.33	1.00
gi348169341	0.02	ND	ND	0.01	0.03	0.05	ND	ND	0.01	0.06	0.50
gi348171839	0.03	ND	ND	ND	0.03	0.04	ND	ND	ND	0.04	0.67
gi348172417	0.06	ND	ND	ND	0.06	0.10	ND	ND	ND	0.10	0.67
gi348174018	0.05	0.74	0.04	ND	0.84	0.09	0.03	0.12	ND	0.24	3.44
gi348173115	0.06	0.03	ND	0.03	0.13	0.03	0.03	ND	0.03	0.10	1.33
gi348169509	0.10	0.15	ND	ND	0.24	0.05	0.10	ND	ND	0.15	1.67
gi348170695	0.13	0.43	0.13	0.23	0.93	0.10	0.33	0.23	0.20	0.87	1.08
gi348175500	0.04	0.12	0.13	0.09	0.38	0.05	0.03	0.12	0.11	0.31	1.21
gi348171739	0.15	0.15	ND	ND	0.31	0.31	0.19	ND	ND	0.50	0.62

gi348172844	0.05	0.10	0.05	ND	0.20	0.03	0.10	0.03	ND	0.15	1.33
gi348173194	0.09	ND	ND	ND	0.09	0.04	ND	ND	ND	0.04	2.00
gi348175942	0.04	ND	ND	ND	0.04	0.02	ND	ND	ND	0.02	2.00
gi348174734	0.07	ND	ND	ND	0.07	0.05	ND	ND	ND	0.05	1.33
gi348173933	0.03	0.11	ND	ND	0.15	0.02	0.02	ND	ND	0.05	3.00
gi348176421	0.10	ND	ND	ND	0.10	0.08	ND	ND	ND	0.08	1.33
gi348172212	0.04	0.06	0.06	0.04	0.20	0.05	0.05	0.04	0.02	0.16	1.25
gi348175615	0.05	ND	0.05	0.06	0.16	0.04	ND	0.02	0.04	0.10	1.63
gi348171829	0.13	ND	ND	ND	0.13	0.17	ND	ND	ND	0.17	0.80
gi348170168	0.25	0.38	ND	0.25	0.88	0.19	0.38	ND	0.13	0.69	1.27
gi348173112	0.12	ND	ND	ND	0.12	0.12	ND	ND	ND	0.12	1.00
gi348176416	0.08	ND	ND	ND	0.08	0.06	ND	ND	ND	0.06	1.33
gi348176812	0.11	ND	ND	ND	0.11	0.11	ND	ND	ND	0.11	1.00
gi348175399	0.11	0.17	ND	ND	0.28	0.06	0.08	ND	ND	0.14	2.00
gi348175275	0.10	ND	ND	ND	0.10	0.05	ND	ND	ND	0.05	2.00
gi348176573	0.09	ND	ND	ND	0.09	0.11	ND	ND	ND	0.11	0.80
gi348175476	0.05	0.06	0.03	ND	0.14	0.09	0.06	0.05	ND	0.21	0.69
gi348176805	0.10	0.07	ND	ND	0.17	0.10	0.10	ND	ND	0.20	0.88
gi348175481	0.17	ND	ND	ND	0.17	0.25	ND	ND	ND	0.25	0.67
gi348170348	0.07	0.22	0.04	0.04	0.36	0.11	0.18	0.15	0.07	0.51	0.71
gi348175444	0.27	0.47	ND	ND	0.73	0.53	0.47	ND	ND	1.00	0.73
gi348175025	0.09	ND	ND	ND	0.09	0.14	ND	ND	ND	0.14	0.67
gi348171618	0.21	ND	ND	ND	0.21	0.16	ND	ND	ND	0.16	1.33
gi348175360	0.05	ND	0.05	ND	0.11	0.03	ND	0.03	ND	0.05	2.00
gi348175301	0.12	ND	ND	ND	0.12	0.09	ND	ND	ND	0.09	1.33

gi348172686	0.17	ND	0.13	0.08	0.38	0.08	ND	0.13	0.25	0.46	0.82
gi348170759	0.11	ND	ND	ND	0.11	0.11	ND	ND	ND	0.11	1.00
gi348169862	0.11	0.11	ND	ND	0.23	0.09	0.20	ND	ND	0.29	0.80
gi348174619	0.27	0.27	ND	ND	0.53	0.33	0.33	ND	ND	0.67	0.80
gi348171969	0.25	ND	ND	ND	0.25	0.13	ND	ND	ND	0.13	2.00
gi348173254	0.07	ND	ND	ND	0.07	0.03	ND	ND	ND	0.03	2.00
gi348175962	0.33	ND	ND	ND	0.33	0.25	ND	ND	ND	0.25	1.33
gi348174801	0.03	0.02	ND	ND	0.04	0.03	0.02	ND	ND	0.05	0.83
gi348174999	0.09	0.20	0.69	0.17	1.14	0.26	0.14	0.80	0.31	1.51	0.75
gi348173901	0.10	ND	ND	ND	0.10	0.10	ND	ND	ND	0.10	1.00
gi348173921	0.02	0.04	ND	ND	0.06	0.04	0.06	ND	ND	0.10	0.60
gi348174862	0.01	0.04	ND	ND	0.05	0.01	0.03	ND	ND	0.04	1.33
gi348173494	0.04	0.08	ND	ND	0.13	0.03	0.06	ND	ND	0.08	1.50
gi348175410	0.02	0.01	ND	ND	0.03	0.03	0.03	ND	ND	0.06	0.56
gi348169304	0.05	0.15	0.11	ND	0.31	0.16	0.07	0.04	ND	0.27	1.13
gi348169399	0.02	0.05	ND	ND	0.07	0.02	0.02	ND	ND	0.04	1.80
gi348176801	0.03	ND	ND	ND	0.03	0.05	ND	ND	ND	0.05	0.60
gi348169196	0.06	0.08	0.06	ND	0.19	0.08	0.08	0.06	ND	0.21	0.91
gi348169427	0.04	ND	ND	ND	0.04	0.02	ND	ND	ND	0.02	1.50
gi348174401	0.02	0.09	0.01	ND	0.13	0.06	0.06	0.01	ND	0.13	0.94
gi348174183	0.04	ND	ND	ND	0.04	0.03	ND	ND	ND	0.03	1.50
gi348174017	0.05	ND	ND	ND	0.05	0.07	ND	ND	ND	0.07	0.75
gi348169182	0.05	0.05	ND	ND	0.09	0.05	0.08	ND	ND	0.12	0.75
gi348175111	0.08	ND	ND	ND	0.08	0.13	ND	ND	ND	0.13	0.60
gi348172915	0.30	0.40	ND	0.30	1.00	0.50	0.60	ND	0.20	1.30	0.77

gi348169559	0.09	ND	ND	ND	0.09	0.17	ND	ND	ND	0.17	0.50
gi348174033	0.19	0.25	0.13	ND	0.56	0.25	0.38	0.13	ND	0.75	0.75
gi348173524	0.16	0.26	ND	ND	0.42	0.21	0.58	ND	ND	0.79	0.53
gi348173156	0.06	ND	ND	ND	0.06	0.06	ND	ND	ND	0.06	1.00
gi348173376	0.05	ND	ND	ND	0.05	0.06	ND	ND	ND	0.06	0.75
gi348176410	0.05	ND	ND	ND	0.05	0.04	ND	ND	ND	0.04	1.50
gi348169423	0.06	0.11	ND	ND	0.17	0.04	0.08	ND	ND	0.11	1.50
gi348173516	0.05	ND	ND	ND	0.05	0.03	ND	ND	ND	0.03	1.50
gi348171861	0.03	ND	ND	ND	0.03	0.03	ND	ND	ND	0.03	0.75
gi348171815	0.04	0.05	ND	ND	0.09	0.04	0.06	ND	ND	0.10	0.87
gi348172073	0.18	ND	ND	ND	0.18	0.18	ND	ND	ND	0.18	1.00
gi348174600	0.12	ND	ND	ND	0.12	0.20	ND	ND	ND	0.20	0.60
gi348175308	0.07	ND	0.12	ND	0.19	0.12	ND	0.07	ND	0.19	1.00
gi348170889	0.06	0.12	ND	ND	0.18	0.04	0.16	ND	ND	0.20	0.90
gi348172514	0.05	ND	ND	ND	0.05	0.03	ND	ND	ND	0.03	1.50
gi348171154	0.13	0.38	0.58	ND	1.08	0.29	0.29	0.50	ND	1.08	1.00
gi348171386	0.06	ND	ND	ND	0.06	0.04	ND	ND	ND	0.04	1.50
gi348174856	0.04	0.10	ND	ND	0.15	0.03	0.03	ND	ND	0.06	2.50
gi348173217	0.12	ND	ND	ND	0.12	0.12	ND	ND	ND	0.12	1.00
gi348169130	0.06	0.16	ND	ND	0.22	0.06	0.08	ND	ND	0.14	1.57
gi348169493	0.06	ND	ND	ND	0.06	0.06	ND	ND	ND	0.06	1.00
gi348176774	0.07	ND	ND	ND	0.07	0.07	ND	ND	ND	0.07	1.00
gi348176688	0.07	0.09	ND	ND	0.16	0.05	0.11	ND	ND	0.16	1.00
gi348176913	0.11	ND	ND	ND	0.11	0.15	ND	ND	ND	0.15	0.75
gi348171181	0.09	0.24	ND	0.12	0.45	0.09	0.15	ND	0.06	0.30	1.50

gi41350154	0.16	ND	ND	ND	0.16	0.11	ND	ND	ND	0.11	1.50
gi348173013	0.14	ND	0.14	0.09	0.36	0.23	ND	0.18	0.14	0.55	0.67
gi348175006	0.19	ND	ND	ND	0.19	0.19	ND	ND	ND	0.19	1.00
gi348175348	0.15	ND	ND	ND	0.15	0.15	ND	ND	ND	0.15	1.00
gi348171621	0.06	0.04	ND	ND	0.10	0.06	0.04	ND	ND	0.10	1.00
gi348175714	0.08	0.08	ND	ND	0.16	0.08	0.16	ND	ND	0.24	0.67
gi348170905	0.30	ND	ND	ND	0.30	0.30	ND	ND	ND	0.30	1.00
gi348170087	0.30	0.80	ND	ND	1.10	0.60	0.60	ND	ND	1.20	0.92
gi348169138	0.06	0.04	ND	ND	0.09	0.04	0.04	ND	ND	0.08	1.25
gi348172470	0.38	0.63	2.13	ND	3.13	0.25	0.38	2.63	ND	3.25	0.96
gi348171760	0.07	ND	0.07	ND	0.15	0.07	ND	0.10	ND	0.17	0.86
gi348176513	0.06	0.04	ND	ND	0.10	0.08	0.04	ND	ND	0.13	0.83
gi348176386	0.01	ND	ND	ND	0.01	0.01	ND	ND	ND	0.01	0.67
gi348169048	0.02	0.04	ND	ND	0.06	0.02	0.04	ND	ND	0.06	1.00
gi348170950	0.03	ND	ND	ND	0.03	0.03	ND	ND	ND	0.03	1.00
gi348171475	0.01	ND	ND	ND	0.01	0.02	ND	ND	ND	0.02	0.67
gi348169250	0.03	ND	ND	ND	0.03	0.03	ND	ND	ND	0.03	1.00
gi348170155	0.02	0.15	ND	0.02	0.19	0.08	0.08	ND	0.05	0.20	0.94
gi348173729	0.02	ND	ND	ND	0.02	0.04	ND	ND	ND	0.04	0.50
gi348171483	0.04	ND	ND	ND	0.04	0.04	ND	ND	ND	0.04	1.00
gi348176681	0.02	ND	ND	ND	0.02	0.02	ND	ND	ND	0.02	1.00
gi348169184	0.02	ND	ND	ND	0.02	0.03	ND	ND	ND	0.03	0.67
gi348169366	0.02	ND	ND	ND	0.02	0.02	ND	ND	ND	0.02	1.00
gi348172987	0.07	0.14	ND	ND	0.21	0.10	0.17	ND	ND	0.28	0.75
gi348176493	0.04	ND	ND	ND	0.04	0.04	ND	ND	ND	0.04	1.00

gi348173037	0.03	ND	ND	ND	0.03	0.06	ND	ND	ND	0.06	0.50
gi348174196	0.06	ND	ND	ND	0.06	0.06	ND	ND	ND	0.06	1.00
gi348169478	0.04	0.09	ND	ND	0.13	0.11	0.16	ND	ND	0.27	0.47
gi348175427	0.02	ND	ND	ND	0.02	0.02	ND	ND	ND	0.02	1.00
gi348174848	0.04	0.07	ND	ND	0.11	0.05	0.04	ND	ND	0.09	1.20
gi348175786	0.05	ND	ND	ND	0.05	0.05	ND	ND	ND	0.05	1.00
gi348169411	0.05	ND	ND	ND	0.05	0.07	ND	ND	ND	0.07	0.67
gi348168994	0.12	1.12	0.35	0.29	1.88	0.29	1.18	0.41	0.29	2.18	0.86
gi348176427	0.05	ND	ND	ND	0.05	0.10	ND	ND	ND	0.10	0.50
gi348169468	0.03	ND	ND	ND	0.03	0.05	ND	ND	ND	0.05	0.50
gi348175266	0.03	ND	ND	ND	0.03	0.03	ND	ND	ND	0.03	1.00
gi348174422	0.04	0.04	ND	ND	0.08	0.06	0.06	ND	ND	0.11	0.67
gi348171421	0.02	ND	ND	ND	0.02	0.05	ND	ND	ND	0.05	0.50
gi348171470	0.15	0.31	ND	ND	0.46	0.31	0.15	ND	ND	0.46	1.00
gi348172068	0.02	ND	ND	ND	0.02	0.02	ND	ND	ND	0.02	1.00
gi348170154	0.03	ND	ND	ND	0.03	0.06	ND	ND	ND	0.06	0.50
gi348176888	0.03	0.03	ND	ND	0.06	0.04	0.08	ND	ND	0.13	0.44
gi348169075	0.04	ND	ND	ND	0.04	0.06	ND	ND	ND	0.06	0.67
gi348173610	0.04	ND	ND	ND	0.04	0.04	ND	ND	ND	0.04	1.00
gi348170978	0.04	ND	ND	ND	0.04	0.07	ND	ND	ND	0.07	0.67
gi348174850	0.03	0.06	0.05	ND	0.14	0.03	0.03	0.03	ND	0.09	1.50
gi348172575	0.02	ND	ND	ND	0.02	0.02	ND	ND	ND	0.02	1.00
gi348174847	0.11	ND	ND	ND	0.11	0.11	ND	ND	ND	0.11	1.00
gi348171464	0.07	0.14	ND	ND	0.21	0.07	0.18	ND	ND	0.25	0.86
gi348174188	0.18	0.18	0.27	ND	0.64	0.27	0.27	0.18	ND	0.73	0.88

gi348175009	0.08	0.21	0.08	ND	0.38	0.21	0.29	0.13	ND	0.63	0.60
gi348173303	0.04	ND	ND	ND	0.04	0.04	ND	ND	ND	0.04	1.00
gi348175005	0.06	ND	ND	ND	0.06	0.12	ND	ND	ND	0.12	0.50
gi348174959	0.03	ND	ND	ND	0.03	0.05	ND	ND	ND	0.05	0.50
gi348170606	0.05	ND	ND	ND	0.05	0.05	ND	ND	ND	0.05	1.00
gi348176150	0.03	ND	ND	ND	0.03	0.05	ND	ND	ND	0.05	0.67
gi348169323	0.03	ND	ND	ND	0.03	0.03	ND	ND	ND	0.03	1.00
gi348172629	0.09	ND	ND	ND	0.09	0.09	ND	ND	ND	0.09	1.00
gi348169303	0.04	ND	ND	ND	0.04	0.08	ND	ND	ND	0.08	0.50
gi348169292	0.05	0.09	ND	ND	0.14	0.05	0.05	ND	ND	0.09	1.50
gi348173898	0.02	ND	ND	ND	0.02	0.04	ND	ND	ND	0.04	0.40
gi348176720	0.13	ND	ND	ND	0.13	0.13	ND	ND	ND	0.13	1.00
gi348176391	0.03	ND	ND	ND	0.03	0.03	ND	ND	ND	0.03	1.00
gi348176950	0.11	ND	ND	ND	0.11	0.11	ND	ND	ND	0.11	1.00
gi348171656	0.02	ND	ND	0.02	0.04	0.03	ND	ND	0.02	0.05	0.80
gi348174639	0.03	0.07	ND	ND	0.10	0.03	0.04	ND	ND	0.07	1.40
gi348169816	0.10	0.20	0.10	0.15	0.55	0.10	0.20	0.10	0.25	0.65	0.85
gi348176472	0.05	ND	ND	ND	0.05	0.05	ND	ND	ND	0.05	1.00
gi348170553	0.10	ND	ND	ND	0.10	0.10	ND	ND	ND	0.10	1.00
gi348169762	0.20	ND	ND	ND	0.20	0.40	ND	ND	ND	0.40	0.50
gi348170112	0.14	ND	0.14	ND	0.29	0.14	ND	0.21	ND	0.36	0.80
gi348174970	0.22	ND	ND	ND	0.22	0.44	ND	ND	ND	0.44	0.50
gi348172418	0.03	ND	ND	ND	0.03	0.05	ND	ND	ND	0.05	0.67
gi348170663	0.10	ND	ND	ND	0.10	0.15	ND	ND	ND	0.15	0.67
gi348170246	0.04	0.28	0.09	ND	0.41	0.04	0.17	0.04	ND	0.26	1.58

gi348168933	0.11	ND	ND	ND	0.11	0.11	ND	ND	ND	0.11	1.00
gi348170891	0.04	ND	ND	ND	0.04	0.05	ND	ND	ND	0.05	0.67
gi348171411	0.03	0.08	0.06	0.05	0.23	0.03	0.06	0.05	0.03	0.18	1.27
gi348176889	0.04	0.10	ND	ND	0.14	0.04	0.12	ND	ND	0.16	0.88
gi348175191	0.05	0.19	0.11	0.05	0.41	0.05	0.08	0.11	0.14	0.38	1.07
gi348174143	0.22	ND	ND	ND	0.22	0.22	ND	ND	ND	0.22	1.00
gi348168923	0.07	ND	ND	ND	0.07	0.10	ND	ND	ND	0.10	0.67
gi348170898	0.09	0.09	0.23	ND	0.41	0.09	0.09	0.18	ND	0.36	1.13
gi348169093	0.15	ND	ND	ND	0.15	0.23	ND	ND	ND	0.23	0.67
gi348174960	0.03	0.04	0.03	ND	0.09	0.03	0.04	0.03	ND	0.09	1.00
gi348175212	0.03	0.04	ND	ND	0.07	0.03	0.03	ND	ND	0.06	1.25
gi348172422	0.04	ND	ND	ND	0.04	0.05	ND	ND	ND	0.05	0.67
gi348175326	0.18	ND	ND	ND	0.18	0.18	ND	ND	ND	0.18	1.00
gi348172330	0.07	ND	ND	ND	0.07	0.17	ND	ND	ND	0.17	0.40
gi348173021	0.17	ND	ND	ND	0.17	0.17	ND	ND	ND	0.17	1.00
gi348172486	0.02	ND	ND	ND	0.02	0.03	ND	ND	ND	0.03	0.67
gi348168939	0.13	0.20	ND	ND	0.33	0.13	0.13	ND	ND	0.27	1.25
gi348169600	0.03	ND	ND	ND	0.03	0.05	ND	ND	ND	0.05	0.67
gi348174322	ND	0.57	1.51	1.67	3.75	ND	0.46	1.74	1.61	3.81	0.98
gi348174073	ND	0.36	0.27	ND	0.64	ND	0.20	0.11	ND	0.31	2.04
gi348174398	ND	0.37	ND	ND	0.37	ND	0.32	ND	ND	0.32	1.15
gi348174441	ND	0.23	ND	ND	0.23	ND	0.28	ND	ND	0.28	0.85
gi348174427	ND	0.33	ND	ND	0.33	ND	0.34	ND	ND	0.34	0.95
gi348171388	ND	0.20	0.04	ND	0.24	ND	0.04	0.05	ND	0.10	2.44
gi348175578	ND	0.35	0.04	ND	0.38	ND	0.25	0.08	ND	0.33	1.18

gi348174443	ND	0.16	0.04	ND	0.19	ND	0.10	0.04	ND	0.14	1.40
gi348169788	ND	0.88	ND	ND	0.88	ND	1.47	ND	ND	1.47	0.60
gi348175259	ND	0.08	0.02	ND	0.10	ND	0.05	0.02	ND	0.07	1.50
gi348171497	ND	0.15	ND	0.15	0.31	ND	0.11	ND	0.28	0.39	0.79
gi348171177	ND	0.32	0.91	1.06	2.29	ND	0.38	0.38	1.09	1.85	1.24
gi348175493	ND	0.29	ND	0.09	0.37	ND	0.14	ND	0.09	0.23	1.63
gi348175789	ND	0.24	ND	ND	0.24	ND	0.10	ND	ND	0.10	2.50
gi13443724	ND	0.01	ND	ND	0.01	ND	0.01	ND	ND	0.01	0.89
gi348170633	ND	0.22	ND	0.08	0.30	ND	0.30	ND	0.05	0.35	0.85
gi348175811	ND	0.18	0.04	ND	0.22	ND	0.24	0.09	ND	0.33	0.67
gi348173410	ND	0.15	ND	ND	0.15	ND	0.13	ND	ND	0.13	1.14
gi348174946	ND	0.35	0.35	0.30	1.00	ND	0.43	0.39	0.13	0.96	1.05
gi348176202	ND	0.10	0.09	0.05	0.24	ND	0.10	0.09	0.05	0.24	1.00
gi348171256	ND	0.05	ND	0.01	0.06	ND	0.05	ND	0.01	0.07	0.90
gi348176712	ND	0.14	ND	ND	0.14	ND	0.27	ND	ND	0.27	0.54
gi348169116	ND	0.14	ND	ND	0.14	ND	0.06	ND	ND	0.06	2.33
gi348175407	ND	0.09	0.07	ND	0.16	ND	0.08	0.08	ND	0.16	1.00
gi348169280	ND	0.18	ND	ND	0.18	ND	0.16	ND	ND	0.16	1.17
gi348169669	ND	0.12	0.12	0.21	0.45	ND	0.10	0.05	0.33	0.48	0.93
gi348172929	ND	0.18	ND	ND	0.18	ND	0.12	ND	ND	0.12	1.50
gi348169571	ND	0.04	ND	ND	0.04	ND	0.03	ND	ND	0.03	1.50
gi348176522	ND	0.09	ND	ND	0.09	ND	0.03	ND	ND	0.03	3.00
gi348174447	ND	0.08	ND	ND	0.08	ND	0.03	ND	ND	0.03	3.00
gi348175480	ND	0.10	ND	ND	0.10	ND	0.03	ND	ND	0.03	3.00
gi348174072	ND	0.14	0.14	0.07	0.35	ND	0.05	0.30	0.09	0.44	0.79

gi348175152	ND	0.25	ND	ND	0.25	ND	0.08	ND	ND	0.08	3.00
gi348170279	ND	0.11	ND	ND	0.11	ND	0.09	ND	ND	0.09	1.20
gi348174008	ND	0.10	ND	ND	0.10	ND	0.03	ND	ND	0.03	3.00
gi348171327	ND	0.13	ND	ND	0.13	ND	0.11	ND	ND	0.11	1.20
gi41350157	ND	0.01	ND	ND	0.01	ND	0.01	ND	ND	0.01	0.83
gi13162634	ND	0.01	ND	ND	0.01	ND	0.01	ND	ND	0.01	0.71
gi348171762	ND	0.06	ND	ND	0.06	ND	0.08	ND	ND	0.08	0.71
gi348170590	ND	0.07	ND	ND	0.07	ND	0.03	ND	ND	0.03	2.50
gi348173935	ND	0.03	ND	ND	0.03	ND	0.05	ND	ND	0.05	0.63
gi348174974	ND	0.12	0.05	ND	0.17	ND	0.07	0.05	ND	0.12	1.40
gi348174715	ND	0.07	ND	ND	0.07	ND	0.03	ND	ND	0.03	2.50
gi348170555	ND	0.04	ND	ND	0.04	ND	0.02	ND	ND	0.02	2.50
gi348176267	ND	0.06	0.05	0.03	0.14	ND	0.06	0.05	0.04	0.15	0.92
gi348175498	ND	0.24	ND	0.29	0.52	ND	0.14	ND	0.10	0.24	2.20
gi348176049	ND	0.15	ND	ND	0.15	ND	0.06	ND	ND	0.06	2.50
gi348174853	ND	0.45	0.45	ND	0.91	ND	1.09	0.45	ND	1.55	0.59
gi348172595	ND	0.33	ND	0.13	0.47	ND	0.27	ND	0.20	0.47	1.00
gi348172023	ND	0.02	ND	ND	0.02	ND	0.02	ND	ND	0.02	1.33
gi348169456	ND	0.04	ND	ND	0.04	ND	0.02	ND	ND	0.02	2.00
gi348174832	ND	0.06	ND	ND	0.06	ND	0.13	ND	ND	0.13	0.44
gi348172827	ND	0.04	ND	ND	0.04	ND	0.04	ND	ND	0.04	1.00
gi348171488	ND	0.16	ND	ND	0.16	ND	0.16	ND	ND	0.16	1.00
gi348176838	ND	0.03	ND	ND	0.03	ND	0.05	ND	ND	0.05	0.67
gi348171517	ND	0.06	ND	ND	0.06	ND	0.06	ND	ND	0.06	1.00
gi348175720	ND	0.07	ND	0.04	0.11	ND	0.04	ND	0.04	0.07	1.50

gi348174420	ND	0.06	ND	ND	0.06	ND	0.11	ND	ND	0.11	0.57
gi348173014	ND	0.05	ND	ND	0.05	ND	0.02	ND	ND	0.02	2.00
gi348171067	ND	0.20	ND	ND	0.20	ND	0.15	ND	ND	0.15	1.33
gi348174887	ND	0.07	ND	ND	0.07	ND	0.05	ND	ND	0.05	1.33
gi348173941	ND	0.13	0.10	ND	0.23	ND	0.07	0.07	ND	0.13	1.75
gi348172406	ND	0.02	0.06	0.06	0.14	ND	0.06	0.02	0.05	0.13	1.13
gi348176978	ND	0.04	ND	ND	0.04	ND	0.03	ND	ND	0.03	1.50
gi348175495	ND	0.09	ND	ND	0.09	ND	0.06	ND	ND	0.06	1.50
gi348169385	ND	0.02	ND	ND	0.02	ND	0.01	ND	ND	0.01	1.50
gi348175208	ND	0.16	ND	ND	0.16	ND	0.21	ND	ND	0.21	0.75
gi348169496	ND	0.04	ND	0.04	0.09	ND	0.10	ND	0.06	0.16	0.55
gi348169071	ND	0.05	ND	ND	0.05	ND	0.05	ND	ND	0.05	1.00
gi348172451	ND	0.03	ND	ND	0.03	ND	0.02	ND	ND	0.02	1.50
gi348172772	ND	0.03	0.06	ND	0.09	ND	0.02	0.03	ND	0.06	1.60
gi348174230	ND	0.03	0.02	ND	0.05	ND	0.02	0.02	ND	0.04	1.25
gi348171446	ND	0.06	ND	ND	0.06	ND	0.16	ND	ND	0.16	0.38
gi348170169	ND	0.03	ND	ND	0.03	ND	0.02	ND	ND	0.02	1.50
gi348174981	ND	0.05	ND	ND	0.05	ND	0.03	ND	ND	0.03	1.50
gi348170192	ND	0.04	ND	ND	0.04	ND	0.04	ND	ND	0.04	1.00
gi348173493	ND	0.06	ND	ND	0.06	ND	0.15	ND	ND	0.15	0.43
gi348173172	ND	0.10	ND	ND	0.10	ND	0.10	ND	ND	0.10	1.00
gi348174995	ND	0.09	ND	ND	0.09	ND	0.18	ND	ND	0.18	0.50
gi15077645	ND	0.07	ND	ND	0.07	ND	0.11	ND	ND	0.11	0.60
gi348169535	ND	0.06	ND	ND	0.06	ND	0.12	ND	ND	0.12	0.50
gi348172882	ND	0.08	0.05	ND	0.14	ND	0.14	0.11	ND	0.24	0.56

gi348169797	ND	0.09	ND	0.09	0.19	ND	0.06	ND	0.09	0.16	1.20
gi348170394	ND	0.05	ND	ND	0.05	ND	0.11	ND	ND	0.11	0.40
gi348175892	ND	0.06	ND	ND	0.06	ND	0.12	ND	ND	0.12	0.50
gi348172766	ND	0.01	0.01	ND	0.02	ND	0.02	0.01	ND	0.03	0.57
gi348174195	ND	0.01	ND	ND	0.01	ND	0.02	ND	ND	0.02	0.67
gi348172988	ND	0.01	ND	ND	0.01	ND	0.01	ND	ND	0.01	1.00
gi348172327	ND	0.07	ND	0.07	0.14	ND	0.14	ND	0.07	0.21	0.67
gi348170151	ND	0.01	ND	0.01	0.03	ND	0.02	ND	0.01	0.03	0.80
gi348176568	ND	0.02	ND	ND	0.02	ND	0.02	ND	ND	0.02	1.00
gi348173989	ND	0.03	ND	ND	0.03	ND	0.05	ND	ND	0.05	0.50
gi348172906	ND	0.03	0.03	0.06	0.12	ND	0.05	0.03	0.03	0.11	1.14
gi348168954	ND	0.03	ND	ND	0.03	ND	0.03	ND	ND	0.03	1.00
gi348169606	ND	0.05	0.08	ND	0.13	ND	0.05	0.05	ND	0.11	1.25
gi348175088	ND	0.06	ND	ND	0.06	ND	0.17	ND	ND	0.17	0.33
gi348172216	ND	0.02	0.02	ND	0.05	ND	0.04	0.07	ND	0.11	0.44
gi348176694	ND	0.05	ND	ND	0.05	ND	0.05	ND	ND	0.05	1.00
gi348176691	ND	0.03	ND	ND	0.03	ND	0.03	ND	ND	0.03	1.00
gi348176910	ND	0.06	ND	ND	0.06	ND	0.09	ND	ND	0.09	0.67
gi348173149	ND	0.05	ND	ND	0.05	ND	0.05	ND	ND	0.05	1.00
gi348172446	ND	0.05	ND	ND	0.05	ND	0.05	ND	ND	0.05	1.00
gi348175501	ND	0.03	ND	ND	0.03	ND	0.03	ND	ND	0.03	1.00
gi348175822	ND	0.11	ND	ND	0.11	ND	0.28	ND	ND	0.28	0.40
gi348170085	ND	0.17	ND	ND	0.17	ND	0.42	ND	ND	0.42	0.40
gi348174325	ND	0.07	ND	0.07	0.14	ND	0.07	ND	0.07	0.14	1.00
gi348170680	ND	0.04	ND	0.04	0.07	ND	0.04	ND	0.07	0.11	0.67

gi348170646	ND	0.03	ND	ND	0.03	ND	0.10	ND	ND	0.10	0.33
gi348171938	ND	0.04	ND	0.05	0.09	ND	0.09	ND	0.05	0.15	0.63
gi348170134	ND	0.12	ND	ND	0.12	ND	0.35	ND	ND	0.35	0.33
gi348171885	ND	0.08	0.13	0.08	0.29	ND	0.13	0.13	0.08	0.33	0.88
gi348169129	ND	0.06	ND	ND	0.06	ND	0.12	ND	ND	0.12	0.50
gi348169328	ND	0.05	ND	ND	0.05	ND	0.08	ND	ND	0.08	0.67
gi348170184	ND	0.03	ND	ND	0.03	ND	0.03	ND	ND	0.03	1.00
gi348175263	ND	0.03	ND	ND	0.03	ND	0.05	ND	ND	0.05	0.67
gi348169764	ND	0.10	ND	0.10	0.20	ND	0.10	ND	0.10	0.20	1.00
gi348170412	ND	0.12	ND	ND	0.12	ND	0.24	ND	ND	0.24	0.50
gi348173306	ND	0.02	0.02	ND	0.04	ND	0.02	0.04	ND	0.06	0.67
gi348176727	ND	0.08	ND	ND	0.08	ND	0.08	ND	ND	0.08	1.00
gi348174252	ND	0.06	ND	ND	0.06	ND	0.13	ND	ND	0.13	0.50
gi348169421	ND	0.04	ND	ND	0.04	ND	0.06	ND	ND	0.06	0.67
gi348171479	ND	0.05	ND	ND	0.05	ND	0.07	ND	ND	0.07	0.67
gi348175306	ND	0.04	0.06	ND	0.10	ND	0.08	0.04	ND	0.12	0.83
gi348171343	ND	0.04	ND	ND	0.04	ND	0.04	ND	ND	0.04	1.00
gi348171400	ND	0.04	ND	ND	0.04	ND	0.04	ND	ND	0.04	1.00
gi348169181	ND	0.13	ND	ND	0.13	ND	0.19	ND	ND	0.19	0.67
gi348174810	ND	ND	1.14	5.10	6.24	ND	ND	1.38	4.14	5.52	1.13
gi348173379	ND	ND	0.11	0.06	0.18	ND	ND	0.07	0.14	0.21	0.85
gi348175187	ND	ND	0.20	ND	0.20	ND	ND	0.09	ND	0.09	2.25
gi348173035	ND	ND	0.13	0.09	0.22	ND	ND	0.07	0.11	0.17	1.25
gi348175862	ND	ND	0.06	ND	0.06	ND	ND	0.05	ND	0.05	1.25
gi348172037	ND	ND	0.02	0.02	0.04	ND	ND	0.01	0.01	0.02	1.80

gi348171788	ND	ND	0.14	0.22	0.35	ND	ND	0.14	0.16	0.30	1.18
gi348176649	ND	ND	0.06	0.11	0.17	ND	ND	0.10	0.16	0.25	0.69
gi348171261	ND	ND	0.24	0.41	0.65	ND	ND	0.12	0.12	0.24	2.75
gi348173084	ND	ND	0.09	0.05	0.14	ND	ND	0.07	0.16	0.23	0.60
gi348169308	ND	ND	0.18	0.32	0.50	ND	ND	0.09	0.27	0.36	1.38
gi348176088	ND	ND	0.01	ND	0.01	ND	ND	0.02	ND	0.02	0.50
gi348174967	ND	ND	0.05	ND	0.05	ND	ND	0.08	ND	0.08	0.60
gi348175283	ND	ND	0.06	0.04	0.10	ND	ND	0.04	0.06	0.10	1.00
gi348176499	ND	ND	0.05	ND	0.05	ND	ND	0.03	ND	0.03	1.50
gi348175218	ND	ND	0.06	ND	0.06	ND	ND	0.04	ND	0.04	1.50
gi348175175	ND	ND	0.02	ND	0.02	ND	ND	0.02	ND	0.02	1.00
gi348171164	ND	ND	0.02	ND	0.02	ND	ND	0.02	ND	0.02	0.67
gi348173310	ND	ND	0.05	ND	0.05	ND	ND	0.05	ND	0.05	1.00
gi348171530	ND	ND	0.04	ND	0.04	ND	ND	0.04	ND	0.04	1.00
gi348176883	ND	ND	0.03	0.04	0.06	ND	ND	0.03	0.03	0.05	1.25
gi348173225	ND	ND	0.02	0.02	0.03	ND	ND	0.02	0.02	0.03	1.00
gi348170280	ND	ND	0.04	ND	0.04	ND	ND	0.06	ND	0.06	0.67
gi348170334	ND	ND	0.06	ND	0.06	ND	ND	0.06	ND	0.06	1.00
gi348176732	ND	ND	0.13	0.27	0.40	ND	ND	0.13	0.20	0.33	1.20
gi348173573	ND	ND	0.05	ND	0.05	ND	ND	0.05	ND	0.05	1.00
gi348171383	ND	ND	0.05	ND	0.05	ND	ND	0.05	ND	0.05	1.00
gi348170471	ND	ND	ND	0.17	0.17	ND	ND	ND	0.07	0.07	2.25
gi348171072	ND	ND	ND	0.05	0.05	ND	ND	ND	0.07	0.07	0.83
gi348173105	ND	ND	ND	0.04	0.04	ND	ND	ND	0.03	0.03	1.25
gi348168932	ND	ND	ND	0.13	0.13	ND	ND	ND	0.13	0.13	1.00

gi348171505	ND	ND	ND	0.05	0.05	ND	ND	ND	0.05	0.05	1.00
gi348170466	ND	ND	ND	0.57	0.57	ND	ND	ND	0.71	0.71	0.80
gi348172409	ND	ND	ND	0.31	0.31	ND	ND	ND	0.15	0.15	2.00
gi348176339	ND	ND	ND	0.09	0.09	ND	ND	ND	0.09	0.09	1.00
gi348174071	ND	ND	ND	0.03	0.03	ND	ND	ND	0.04	0.04	0.75
gi13162642	ND	ND	ND	0.03	0.03	ND	ND	ND	0.03	0.03	1.00
gi348175585	ND	ND	ND	0.04	0.04	ND	ND	ND	0.04	0.04	1.00
gi348169794	ND	ND	ND	0.10	0.10	ND	ND	ND	0.06	0.06	1.50
gi348171458	ND	ND	ND	0.10	0.10	ND	ND	ND	0.07	0.07	1.50
gi348176457	ND	ND	ND	0.04	0.04	ND	ND	ND	0.06	0.06	0.67
gi348172955	ND	ND	ND	0.01	0.01	ND	ND	ND	0.01	0.01	0.50
gi348174459	ND	ND	ND	0.02	0.02	ND	ND	ND	0.02	0.02	1.00
gi348175505	ND	ND	ND	0.01	0.01	ND	ND	ND	0.02	0.02	0.67
gi348168984	ND	ND	ND	0.02	0.02	ND	ND	ND	0.05	0.05	0.33
gi348171528	ND	ND	ND	0.03	0.03	ND	ND	ND	0.05	0.05	0.67
gi348174599	ND	ND	ND	0.07	0.07	ND	ND	ND	0.13	0.13	0.50
gi348169202	ND	ND	ND	0.02	0.02	ND	ND	ND	0.02	0.02	1.00
gi348170164	ND	ND	ND	0.03	0.03	ND	ND	ND	0.03	0.03	1.00
gi348170226	ND	ND	ND	0.02	0.02	ND	ND	ND	0.03	0.03	0.67
gi348176716	ND	ND	ND	0.04	0.04	ND	ND	ND	0.04	0.04	1.00
gi348171716	ND	ND	ND	0.03	0.03	ND	ND	ND	0.05	0.05	0.50

[a, b] the individual PAI values for each protein from the four time phases were added to determine the ΣPAI_{BR1} and ΣPAI_{BR2} for each of the 689 proteins that were quantified in replicates 1 and 2, respectively. ND, The protein is not detected.

Table S2 The most abundant proteins in *S. spinosa* (PAI > 3)

NCBI NO.	Protein description	Averaged PAI ratios from two biological replicates [a]						∑PAI value [b]	Function and KEGG [c]
		T2/T1	T3/T1	T4/T1	T3/T2	T4/T2	T4/T3		
gi348176726	hypothetical protein SspiN1_40323	4.52	0.71	0.30	0.16	0.07	0.42	140.61	No classification
gi348173178	superoxide dismutase [Fe-Zn] 1 (FeSOD I)	0.97	0.52	0.77	0.53	0.79	1.49	63.52	Peroxisome
gi348173691	cold shock protein CspA	0.91	0.25	0.07	0.27	0.08	0.29	38.42	Transcription factors
gi348175327	hypothetical protein SspiN1_33090	1.31	1.64	1.73	1.25	1.32	1.06	31.80	No classification
gi348176721	hypothetical protein SspiN1_40298	1.73	ND	ND	ND	ND	ND	28.40	No classification
gi348174902	metallopeptidase	1.88	0.84	0.30	0.45	0.16	0.35	23.45	Catabolism/proteases
gi348174397	cold-shock DNA-binding domain-containing protein	0.72	0.21	0.03	0.29	0.04	0.13	21.31	Transcription factors
gi348173102	chaperonin GroEL	0.98	0.19	0.09	0.19	0.09	0.48	18.53	Folding, Sorting and Degradation
gi348172783	hypothetical protein SspiN1_20066	1.37	1.00	0.47	0.73	0.35	0.47	18.25	No classification
gi348173083	serine protease precursor	1.72	0.33	0.21	0.19	0.12	0.63	16.26	Signal transduction
gi348176443	hypothetical protein SspiN1_38884	1.82	0.39	0.29	0.22	0.16	0.73	15.68	No classification
gi348169918	cold-shock DNA-binding domain-containing protein	0.54	0.18	0.10	0.33	0.19	0.58	15.38	Transcription factors
gi348170335	zinc binding alcohol dehydrogenase	0.74	0.13	0.05	0.17	0.07	0.43	15.05	Carbohydrate metabolism
gi348174074	polysaccharide deacetylase	4.13	3.18	1.97	0.77	0.48	0.62	14.35	Carbohydrate metabolism
gi348172319	cold-shock DNA-binding domain-containing protein	0.53	0.25	ND	0.46	ND	ND	13.43	Transcription factors
gi348176852	hypothetical protein SspiN1_40990	1.62	1.88	0.81	1.16	0.50	0.43	12.39	No classification
gi348174864	hypothetical protein SspiN1_30689	0.90	0.44	0.21	0.49	0.23	0.47	12.36	No classification
gi348173100	cold shock protein	0.61	0.04	ND	0.07	ND	ND	12.12	Transcription factors
gi348169183	hypothetical protein SspiN1_01430	0.18	0.10	ND	0.56	ND	ND	12.11	No classification
gi348176428	elongation factor Tu	1.45	0.50	0.35	0.34	0.24	0.70	11.87	Translation
gi348174810	hypothetical protein SspiN1_30419	ND	ND	ND	ND	ND	3.66	11.76	No classification

gi348171511	glutamine synthetase	3.61	1.18	0.99	0.33	0.27	0.84	10.93	Amino acid metabolism
gi348174826	membrane spanning protein	2.23	2.57	2.92	1.15	1.31	1.14	10.53	Cell wall/membrane/septation
gi348176851	hypothetical protein SspiN1_40985	1.47	0.56	0.15	0.38	0.10	0.27	10.44	No classification
gi348171558	hypothetical protein SspiN1_13725	1.79	1.14	0.40	0.64	0.23	0.35	10.11	No classification
gi348173043	CutA1 divalent ion tolerance protein	0.83	0.61	0.24	0.74	0.29	0.40	10.00	Resistance protein
gi348175373	ferritin family protein	2.06	2.29	3.82	1.11	1.86	1.67	8.67	Iron ion transport
gi348173142	secreted protein	1.67	ND	ND	ND	ND	ND	7.94	Membrane transport
gi348174322	hypothetical protein SspiN1_27929	ND	ND	ND	3.16	3.18	1.01	7.56	No classification
gi348170208	hydrolase	1.38	1.33	1.24	0.97	0.90	0.93	7.43	Amino acid metabolism
gi348176809	10 kD chaperonin cpn10	0.30	ND	ND	ND	ND	ND	7.08	Folding, Sorting and Degradation
gi348170064	choloylglycine hydrolase	1.61	2.25	2.33	1.40	1.45	1.04	7.00	Lipid metabolism
gi348176725	hypothetical protein SspiN1_40318	2.25	ND	2.00	ND	0.89	ND	7.00	No classification
gi348169928	hypothetical protein SspiN1_05293	2.27	1.64	1.91	0.72	0.84	1.17	6.82	No classification
gi348170152	lysozyme M1 precursor	1.73	0.16	ND	0.09	ND	ND	6.52	Catabolism/proteases
gi348172470	hypothetical protein SspiN1_18471	1.60	7.60	ND	4.75	ND	ND	6.38	No classification
gi348170524	hypothetical protein SspiN1_08400	0.89	ND	0.13	ND	0.14	ND	6.28	No classification
gi348173909	uroporphyrin-III C/tetrapyrrole (Corrin/Porphyrin) methyltransferase	0.35	ND	ND	ND	ND	ND	6.25	Metabolism of cofactors and vitamins
gi348174563	cyclic nucleotide-binding domain-containing protein	2.40	0.64	0.07	0.27	0.03	0.11	6.24	Binding protein/chaperones
gi348170321	5-methyltetrahydropteroyltriglutamate--homocysteine S-methyltransferase	1.89	0.74	0.58	0.39	0.31	0.78	6.18	Amino acid metabolism
gi348172914	hypothetical protein SspiN1_20739	1.25	ND	ND	ND	ND	ND	6.00	No classification
gi348171612	AhpC/TSA family protein	0.60	0.14	ND	0.23	ND	ND	5.80	Peroxiredoxin
gi348176468	50S ribosomal protein L7/L12	0.72	0.24	ND	0.33	ND	ND	5.52	Translation
gi348175422	PS1 protein	1.50	0.28	0.11	0.19	0.07	0.39	5.52	Translation
gi348176887	oligopeptide binding protein of ABC transporter	1.10	0.88	0.38	0.80	0.34	0.43	5.51	ABC transporter

gi348169435	secreted protein	3.37	ND	ND	ND	ND	ND	5.37	Membrane transport
gi348169092	F0F1 ATP synthase subunit beta	0.80	0.17	0.17	0.22	0.21	0.97	5.33	Energy metabolism
gi348169997	protease	1.00	0.19	ND	0.19	ND	ND	5.09	Catabolism/proteases
gi348173307	NmrA family protein	1.67	1.02	0.14	0.61	0.08	0.14	5.07	Transcriptional regulator
gi348176208	luciferase-like protein	1.86	2.11	2.25	1.13	1.21	1.07	4.93	Oxidoreductase activity
gi348174687	cyclic nucleotide-binding domain-containing protein	2.98	1.36	0.21	0.46	0.07	0.16	4.76	Binding protein /chaperones
gi348172972	alcohol dehydrogenase zinc-binding domain-containing protein	0.97	0.28	0.20	0.29	0.21	0.71	4.49	Carbohydrate metabolism
gi41350153	urease alpha subunit	1.65	1.26	1.20	0.77	0.73	0.95	4.48	Nucleotide metabolism
gi348176722	hypothetical protein SspiN1_40303	1.70	0.36	ND	0.21	ND	ND	4.39	No classification
gi348171262	NAD/factor-dependent formaldehyde dehydrogenase	1.14	0.67	0.48	0.58	0.42	0.72	4.37	Binding protein /chaperones
gi348171298	stress-like protein	2.58	1.38	2.15	0.54	0.84	1.56	4.20	Stress protein
gi348171177	heat shock protein Hsp20	ND	ND	ND	1.83	3.04	1.66	4.15	Folding, Sorting and Degradation
gi348171500	dihydrolipoamide dehydrogenase	2.21	1.07	0.97	0.48	0.44	0.90	4.11	Carbohydrate metabolism
gi348168994	protease inhibitor protein	5.57	1.86	1.43	0.33	0.26	0.77	4.06	Catabolism /proteases
gi348174564	cyclic nucleotide-binding protein	2.02	0.59	0.04	0.29	0.02	0.07	4.03	Binding protein /chaperones
gi348170525	hypothetical protein SspiN1_08405	1.36	0.91	ND	0.67	ND	ND	4.00	No classification
gi348174019	glyceraldehyde 3-phosphate dehydrogenase	1.03	0.26	0.18	0.26	0.18	0.69	3.94	Carbohydrate metabolism
gi348173305	aldo/keto reductase	1.05	0.59	0.10	0.56	0.09	0.17	3.91	Metabolism of terpenoids and polyketides
gi348169336	hypothetical protein SspiN1_02205	1.08	0.62	ND	0.57	ND	ND	3.89	No classification
gi348175040	fructose-bisphosphate aldolase	0.96	0.41	0.25	0.43	0.26	0.62	3.65	Carbohydrate metabolism
gi348174688	terpene synthase, metal-binding protein	1.80	0.67	0.08	0.37	0.04	0.12	3.62	Metabolism of terpenoids and polyketides
gi348174689	methyltransferase	1.62	0.10	ND	0.06	ND	ND	3.59	Metabolism of terpenoids and polyketides
gi348174411	aldehyde dehydrogenase	0.83	0.26	0.32	0.31	0.39	1.23	3.49	Carbohydrate metabolism
gi348175715	polyketide biosynthesis enoyl-CoA hydratase	1.26	0.12	ND	0.10	ND	ND	3.48	Metabolism of terpenoids and polyketides

gi348168991	OsmC family protein	2.10	ND	ND	ND	ND	ND	3.44	Stress protein
gi348171389	ATP-dependent protease Clp, ATPase	0.24	ND	ND	ND	ND	ND	3.40	Catabolism /proteases
gi348172782	hypothetical protein SspiN1_20061	1.67	0.17	ND	0.10	ND	ND	3.27	No classification
gi348171813	ATP-dependent Clp protease proteolytic subunit	1.24	1.03	0.88	0.83	0.71	0.85	3.26	Catabolism /proteases
gi348171412	putative glutamate binding protein	2.93	2.17	1.34	0.74	0.46	0.62	3.22	Transporter
gi348170176	enoyl-(acyl carrier protein) reductase	0.86	0.33	ND	0.39	ND	ND	3.21	Lipid metabolism
gi348175295	hypothetical protein SspiN1_32924	1.11	1.13	0.70	1.02	0.63	0.62	3.19	No classification
gi348172504	luciferase-like protein	1.23	0.15	ND	0.12	ND	ND	3.19	Oxidoreductase activity
gi348175423	PS1 protein	1.95	0.77	0.37	0.39	0.19	0.48	3.14	Translation

[a] Averaged PAI ratios from two biological replicates. T2/T1, T3/T1, T4/T1, T3/T2, T4/T2, and T4/T3 represent the six pairs of comparisons of the average PAI for each protein. ND, The protein is not detected in at least one phase.

[b] ΣPAI is the total PAI of two biological replicates during the four phases.

[c] The functions and KEGG pathways of each protein are classified according to their annotated functions in GenBank as well as based on their homology or function, as described in their gene ontology, conserved domains, and KEGG pathways.

Table S3 Proteins for Spinosad biosynthesis in *S. spinosa*

Function classification [a]	NCBI NO.	Gene [b]	Description	Identified peptides [c]			
				T1	T2	T3	T4
Polyketide biosynthesis	gi348172135	*spnA*	modular polyketide synthase, partial	ND	ND	ND	1
	gi13162637	*spnB*	polyketide synthase extender module 2	8	11	ND	ND
	gi13162638	*spnC*	polyketide synthase extender modules 3-4	ND	3	ND	ND
	gi13162634	*spnE*	polyketide synthase extender modules 8-10	2	6	ND	ND
	gi348175713		modular polyketide synthase	3	1	1	ND
	gi348174739		modular polyketide synthase	ND	ND	1	ND
	gi348175715		polyketide biosynthesis enoyl-CoA hydratase	41	52	5	1
	gi348173393		beta-ketoacyl synthase, partial	ND	ND	1	ND
	gi348173394		beta-ketoacyl synthase, partial	ND	ND	1	ND
	gi348175718		beta-ketoacyl synthase	7	3	ND	ND
	gi348173385		beta-ketoacyl synthase	1	ND	ND	ND
	gi348173390		beta-ketoacyl synthase	ND	ND	ND	1
	gi348177040		beta-ketoacyl synthase	ND	ND	ND	1
	gi348175674		beta keto-acyl synthase	ND	ND	1	ND
	gi348174599		putative acyltransferase	1	1	2	3
	gi348169131		putative acyltransferase	ND	3	ND	ND
	gi348170219		aldo/keto reductase family oxidoreductase	1	ND	ND	ND
	gi348169606		aldo/keto reductase family oxidoreductase	1	2	3	2
	gi348174778		aldo/keto reductase family oxidoreductase	ND	1	ND	ND

Polyketide biosynthesis	gi348175336		aldo/keto reductase family oxidoreductase	ND	ND	1	ND
	gi348173305		aldo/keto reductase	46	48	27	5
	gi348170249		aldo/keto reductase	ND	1	ND	ND
	gi348171622		acyl carrier protein	10	5	ND	ND
	gi348175720		polyketide beta-ketoacyl:acyl carrier protein synthase	ND	3	2	2
	gi348170176		enoyl-(acyl carrier protein) reductase	29	25	10	3
	gi348169040		putative 3-hydroxyacyl-CoA dehydrogenase	7	5	3	1
	gi348171858		thioesterase family protein	ND	1	ND	ND
	gi348169563		acyl-CoA thioesterase II	ND	1	ND	ND
Rhamnose biosynthesis	gi15077647	*gdh*	DTDP-glucose 4,6-dehydratase	ND	1	ND	ND
	gi15077645	*gtt*	putative glucose-1-phosphate thymidyltransferase	2	4	ND	ND
	gi348175864		glycosyl transferase, group 1	ND	2	ND	ND
	gi348174384		glycosyl transferase	1	ND	ND	ND
	gi348174443		glycosyl transferase	ND	14	4	ND
	gi348174728		putative glycosyltransferase	1	ND	ND	ND
	gi348175029		transglycosylase family protein	2	ND	ND	ND
	gi13162641	*spnH*	probable O-methyltransferase	1	1	ND	ND
	gi13162642	*spnI*	probable O-methyltransferase	1	6	2	3
	gi13162644	*spnK*	probable O-methyltransferase	6	11	1	ND
	gi348171103		SAM-dependent methyltransferase	1	ND	ND	ND
	gi348174447		UDP-glucose/GDP-mannose dehydrogenase	ND	4	ND	ND

Forosamine biosynthesis	gi348173856		oxidoreductase domain-containing protein	1	ND	ND	ND
	gi348173408	*spnQ*	lipopolysaccharide biosynthesis protein RfbH	1	ND	ND	ND
	gi13162651	*spnR*	probable aminotransferase	ND	1	ND	ND
	gi348173410	*spnS*	type 11 methyltransferase	1	8	2	ND
	gi348169314		4-aminobutyrate aminotransferase, PLP-dependent	ND	3	2	1
Polyketide bridging	gi348173396	*spnF*	C5-O-methyltransferase	2	2	ND	ND
	gi348173400	*spnJ*	putative oxidoreductase	ND	2	2	1
	gi13162646	*spnM*	SpnM	ND	ND	1	ND
Other proteins	gi348174086		type 11 methyltransferase	ND	1	ND	ND
	gi348174689		methyltransferase	29	47	3	ND
	gi348174734		O-methyltransferase	4	ND	ND	ND
	gi348172815		O-methyltransferase	1	2	1	1
	gi348170053		putative methyltransferase	ND	1	ND	ND
	gi348168946		methyltransferases-like protein	ND	1	ND	ND

[a] The functions of each protein are classified according to their annotated functions in GenBank as well as based on their homology or function, as described in their gene ontology, conserved domains, and KEGG pathways.

[b] Genes have been studied and identified to code corresponding proteins in other researches.

[c] Total number of identified peptides from two biological replicates. ND, The protein is not detected in any phase.

Table S4 Oligonucleotide primers used in this study

Name	Sequence (5'-3')
**(A)PCR primers**	
MHSM-F HindIII	GGGAAGCTTTCAGCGCAGCTCGGCGAT
MHSM-R EcoRI	CCGGAATTCATGACCAGCAGAATCGGATCTACC
GS-F EcoRI	CCGGAATTCATGTTCAAGAATCCTGACGAGG
GS-R HindIII	GGGAAGCTTTCACACGTCGAAGTACAGCGA
CNDP-S EcoRI	CCGGAATTCATGACTGTGACGGATCCGCTCA
CNDP-A HindIII	GGGAAGCTTTCAGCTCTCCCGGCCGAGTT
**(B)qRT-PCR primers**	
RT-MHSM-F	GCGTTGAGGTCGTCCAGC
RT-MHSM-R	TGCCGAGTCCACCCAGAT
RT-GS-F	ATGTCGCTGTGGAAGGACG
RT-GS-R	AGGATGCCGCCGATGTAG
RT-CNDP-F	CCAGGTCGTGCTCATCGC
RT-CNDP-R	GTTCCAGGTGGTCGTTTCG
RT-PSDA-F	GAGCCGCCTTCTCCACCT
RT-PSDA-R	TGACCACCCGCCAGTTCC
RT-spnK-F	CTCCTCGGGCTTGCTCTGG
RT-spnK-R	TGTCGGTGGCTACAACTTCG
RT-16S-F	ACCCTTGCCCTATGTTGCC
RT-16S-R	CCTTCCTCCGAGTTGACCC

Table S5 Enzyme name abbreviations used in this study

NCBI Accession	Description	Abbreviations
gi348176000	glucose-6-phosphate isomerase	GPI
gi348175040	fructose-bisphosphate aldolase	FBPA
gi348174019	glyceraldehyde 3-phosphate dehydrogenase	GAPDH
gi348174018	phosphoglycerate kinase	PGK
gi348174887	phosphoglycerate mutase	PGM
gi348171500	dihydrolipoamide dehydrogenase	E3
gi348176767	succinyl-CoA synthetase subunit beta	B-SCS
gi348170953	fumarate hydratase	FHD
gi348175998	transketolase	TrK
gi348176848	phosphoglucosamine mutase	PGM
gi348174074	polysaccharide deacetylase	PSDA
gi348170064	choloylglycine hydrolase	CH
gi348174826	membrane spanning protein	MSP
gi348176568	cell wall surface anchor family protein	CWSA
gi348173142	secreted protein	SP-H
gi348169435	secreted protein	SP-L
gi348169513	putative substrate-binding transport protein	SBTP
gi348172844	dipeptide ABC transporter substrate-binding protein	DSBP
gi348173080	sugar ABC transporter periplasmic protein	STPP
gi348171106	ABC-2 type transporter	ABC-2
gi348176086	ABC transporter ATP-binding protein	ATP-BP

gi348172772	putative sugar binding secreted protein	SBSP
gi348171412	putative glutamate binding protein	GBP
gi348171411	putative glutamate ABC transporter ATP-binding protein	G ATP-BP
gi348176887	oligopeptide binding protein of ABC transporter	OPBP
gi348175189	phosphate ABC transporter ATP-binding protein	P ATP-BP
gi348168950	putative ABC transport system substrate binding protein	SSBP
gi348176682	ABC transporter substrate-binding protein	SBP
gi348170845	sugar ABC transporter, ATP-binding protein	S ATP-BP
gi348173178	superoxide dismutase [Fe-Zn] 1 (FeSOD I)	FeSOD I
gi348174946	superoxide dismutase, copper/zinc binding protein	Cu/ZnSOD
gi348168941	putative nickel-containing Superoxide dismutase Precursor	NiSOD P
gi348175373	ferritin family protein	FFP
gi348176208	luciferase-like protein	LLP
gi348168991	OsmC family protein	OsmC
gi348170321	5-methyltetrahydropteroyltriglutamate--homocysteine S-methyltransferase	MHSM
gi348171511	glutamine synthetase	GS
